# Photobiota of the Tropical Red Sea: Fatty Acid Profile Analysis and Nutritional Quality Assessments

**DOI:** 10.3390/molecules30030621

**Published:** 2025-01-31

**Authors:** Sarah A. Gozai-Alghamdi, Samir M. Aljbour, Saeed A. Amin, Susana Agustí

**Affiliations:** 1 Biological and Environmental Science and Engineering Division (BESE), Marine Science Program, King Abdullah University of Science and Technology (KAUST), Thuwal 23955-6900, Saudi Arabia; samir.aljbour@bau.edu.jo (S.M.A.); saeed.amin@kaust.edu.sa (S.A.A.); susana.agusti@kaust.edu.sa (S.A.); 2 Department of Biological Sciences, Faculty of Science, University of Jeddah (UJ), Jeddah 21959, Saudi Arabia; 3 Department of Allied Medical Sciences, Zarqa University College, Al-Balqa Applied University (BAU), Al-Salt 19117, Jordan; 4 Department of Biotechnology, College of Science, Taif University, Taif 21944, Saudi Arabia

**Keywords:** omega-3, fatty acids, polyunsaturated fatty acids, cyanobacteria, eukaryotic microalgae, mangrove, macroalgae, corals, jellyfish, Red Sea

## Abstract

Photosynthetic organisms are primary sources of marine-derived molecules, particularly ω3 fatty acids (FAs), which influence the quality of marine foods. It is reported that tropical organisms possess lower FA nutritional quality than those from colder oceans. However, the high biodiversity known for tropical areas may help compensate for this deficiency by producing a high diversity of molecules with nutritional benefits for the ecosystem. Here we addressed this aspect by analyzing the FA profiles of 20 photosynthetic organisms from the salty and warm Red Sea, a biodiversity hot spot, including cyanobacteria, eukaryotic microalgae, macroalgae, mangrove leaves, as well as three selected reef’s photosymbiotic zooxanthellate corals and jellyfish. Using direct transesterification, gas chromatography-mass spectrometry, FA absolute quantification, and nutritional indexes, we evaluated their lipid nutritional qualities. We observed interspecific and strain-specific variabilities in qualities, which the unique environmental conditions of the Red Sea may help to explain. Generally, eukaryotic microalgae exhibited the highest nutritional quality. The previously unanalyzed diatoms *Leyanella* sp. and *Minutocellus* sp. had the highest eicosapentaenoic acid (EPA) contents. The bioprospected Red Sea photobiota exhibited pharmaceutical and nutraceutical potential. By sourcing and quantifying these bioactive compounds, we highlight the untapped rich biodiversity of the Red Sea and showcase opportunities to harness these potentials.

## 1. Introduction

Fatty acids (FAs) are vital molecules for cellular viability, organism health, aquatic food web dynamics, and overall ecosystem stability. Photosynthetic organisms produce FAs, which accumulate in consumer tissues, which influence human diets [1,2]. Analyzing FA profiles is essential for assessing the nutritional quality of lipids and understanding the health of marine ecosystems. Calculating indexes that prioritize FA composition, particularly essential fatty acids (EFAs), is important for the nutritional quality assessment of lipids [3,4,5,6]. Unlike primary producers, humans lack the desaturase enzymes (Δ12 and Δ15) necessary for synthesizing the EFAs, α-linolenic acid (ALA; C18:3ω3) and linoleic acid (LA; C18:2ω6) and thus depend on dietary intake [7]. These two FAs are the precursors for other EFA, such as the ω3 eicosapentaenoic acid (EPA; 20:5ω3) and docosahexaenoic acid (DHA; 22:6ω3), along with ω6 arachidonic acid (ARA; 20:4ω6), crucial for cardiovascular, brain, and mental health [8,9,10,11]. Mangrove leaves, macroalgae, diatoms, and dinoflagellates are recognized as primary sources of high ALA, ARA, EPA, and DHA, respectively [12,13,14].

Nutritional quality indexes, such as the ω6/ω3 ratio, with recommended dietary intakes of 1:1 to 4:1, help balance inflammatory and anti-inflammatory responses [15,16]. Macroalgae and microalgae are recognized for their high ω6 and ω3 contents, respectively [17]. Additional preferred quality indexes include a higher polyunsaturated fatty acid (PUFA)/saturated fatty acid (SFA) ratio and a lower atherogenic index (AI) to support cardiovascular health and reduce atherogenic risks, respectively. Beyond nutritional relevance, marine bioactive FAs offer numerous pharmaceutical benefits, including antibacterial [18,19], anti-inflammatory [20], and antitumor effects [21].

Studies on the FA compositions of tropical marine photosynthetic organisms, such as macroalgae, have revealed nutraceutical and pharmaceutical potentials [22,23,24]. However, the FA profiles and molecular potentials of many other tropical organisms remain untapped. Photosynthetic organisms from warm waters are often considered to have lower FA quality than those in colder oceans. Studies indicate that microalgae grown at higher temperatures show decreased ω3 EFAs contents and increased SFA and ω6 contents [25]. Additionally, tropical Atlantic phytoplankton have lower FA unsaturation and EPA contents than those at higher latitudes [26]. Meta-analysis indicated the lowest DHA contents in tropical primary producers across latitudinal comparisons [27]. Current FA knowledge is still lacking in studies that capture the high biodiversity of tropical photosynthetic biota [22,25]. Each contributes uniquely to the tropical marine food web. Tropical marine ecosystems are characterized by high biodiversity [28,29,30,31], which significantly expands the range of unique genetic sequences and biochemical compounds, enhancing their nutritional and pharmaceutical potentials. A wide range of primary producers increases the likelihood of containing species with unique and beneficial FAs, which are vital for ecosystem health and for advancing human nutrition and medicine [32,33,34].

The tropical Red Sea, known for its coral reefs along the coast, is recognized as a marine biodiversity hotspot [35,36,37]. This diversity is also attributed to its unique ecological conditions—hot, hypersaline, highly transparent, oligotrophic, isolated, and semi-enclosed—which support high levels of endemism and species richness [36]. Although the diversity of phototrophs in the Red Sea was reported for macroalgae [38,39], and microalgae [40,41], the biodiversity of the Red Sea remains underexplored [42]. The context of the extreme environmental conditions of the Red Sea, particularly under the ongoing rapid warming and increased heatwaves [43], all known to influence metabolism [44,45], is critical as FAs that are present in other habitats in high concentrations take on significant importance in such conditions, highlighting the resilience and adaptability of these species. The complex bathymetry of the Red Sea favors a strong interaction between pelagic (open ocean) and coral reef animals for food, sustaining its high biodiversity. Additionally, many reef animals thrive in symbiotic relationships with photosynthetic zooxanthellae, increasing the FA content of invertebrate hosts [46] and raising the potential of sharing these essential nutrients with consumers. Although warm temperatures decrease membrane FA unsaturation [25,26], the unsaturation of membrane FAs is important in the tolerance of the photosynthetic machinery to salt [47], suggesting that organisms from the Red Sea, one of the saltiest seas on earth, must have adapted their unsaturated FA composition to salinity conditions.

Despite the valuable insights provided by existing studies reporting lipids and FAs from photobiota of the Red Sea [45,48,49,50,51,52,53,54,55,56,57,58], most studies are reporting relative composition, and there is a need for a more comprehensive and quantitative understanding of FA profiles across the primary FA producers of the Red Sea. This will help evaluate their nutritional qualities as well as bioprospecting their FA nutraceutical and pharmaceutical potentials.

In this study, we hypothesize that the rich species diversity of the Red Sea photobiota, together with the unique environmental conditions, enhances the variety of FAs, increasing the nutraceutical and pharmaceutical potentials of the organisms from this tropical ecosystem. To test our hypothesis, our analysis included diverse species, representing a snapshot of the biodiversity of the Red Sea. A common factor the selected organisms in this work share is their capacity for primary FA production, either directly or via symbiotic relationships with photosynthetic entities, which synthesize PUFA and other FAs necessary for the host’s health and energy [46,59].

Analyzed organisms include cyanobacteria (*Synechococcus* sp. and *Trichodesmium* sp. during a bloom), eukaryotic microalgae (10 strains, including several diatoms, dinoflagellates, a cryptophyte, and a chlorophyte), macroalgae (Phaeophyta and Chlorophyta), leaves of the gray mangrove *Avicennia marina*, and photosymbiotic zooxanthellate cnidarians, including the hard coral *Acropora hemprichii*, the soft coral *Xenia* sp. and the epibenthic jellyfish *Cassiopea andromeda,* which represent a relevant component of reefs in coastal tropical ecosystems. Photosynthetic symbiotic dinoflagellates (also known as zooxanthellae) hosted by these selected cnidarians are known to contribute to the FA pool of their hosts, supplying EPA, DHA, and other PUFAs [60,61,62]. Several of these organisms were not previously analyzed for their FA content, either globally (i.e., *Leyanella* sp. and *Minutocellus* sp.) or on the scale of the Red Sea (e.g., *Ac. hemprichii*, *Amphidinium* sp., *Cylindrotheca closterium*, and *Cs. andromeda*). The reef-building coral *Ac. hemprichii* is listed as vulnerable on the ICUN Red List of threatened species. On the other hand, we provided quantitative measurements of the FA content in Red Sea organisms that had been analyzed before in the Red Sea reporting relative values (e.g., *Xenia* sp. [57], *Av. marina* [63], and *Tetraselmis* sp. [49,50]). In this study, the absolute FA contents of Red Sea photobiota will help to reveal a quantitative-based nutritional quality index for these organisms and, moreover, to contribute to establishing a baseline dataset of quantitative FA profiles for tropical organisms. This is crucial for assessing the effects of ongoing warming on metabolism as well as harnessing their nutritional and therapeutic potential.

## 2. Results

### 2.1. Interspecific Variations in Fatty Acid Content and Total Lipid Content

**Figure 1:**
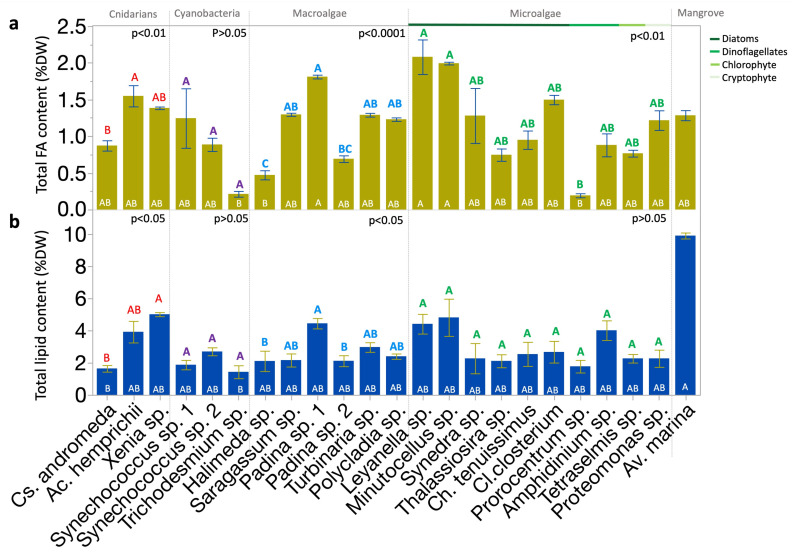
Distribution of total FA and lipid contents across Red Sea photobiota. (**a**) Total content of FA (mean % DW). (**b**) Total content of lipid (mean % DW). Bars show means, with error bars indicating standard error. Statistical analyses: Kruskal–Wallis test followed by the Dunn test, where species ranks that do not share a letter are significantly different. Color-coded letters above the bars correspond to the comparisons within the respective group panel. White letters inside the bars correspond to the comparisons across all species. Replicates from each species and the mean from five biological replicates of leaves of *Av. marina* were used in the comparison. FA, fatty acid; DW, dry weight.

Total FA contents per dry weight (DW) of tropical biota found averaging 9.8 mg g^−1^ DW (range: 1.7–17.8 mg g^−1^ DW). FA content varied significantly across species (*p* < 0.0001; Kruskal–Wallis test; Figure 1a). Interspecific variations were significant within groups (*p* < 0.01 or *p* < 0.0001; Kruskal–Wallis test), except within cyanobacteria (Figure 1a). The organisms analyzed included the macroalgae *Sargassum* sp., *Turbinaria* sp., *Polycladia* sp., *Padina* sp. *1*, *Padina* sp. *2*, and *Halimeda* sp., eukaryotic microalgae—including the diatoms *Cylindrotheca closterium (Cl. Closterium)*, *Thalassiosira* sp., *Synedra* sp., *Chaetoceros tenuissimus (Ch. tenuissimus)*, *Leyanella* sp., and *Minutocellus* sp., the dinoflagellates *Amphidinium* sp. and *Prorocentrum* sp., the cryptophyte *Proteomonas* sp. and the chlorophyte *Tetraselmis* sp. Additionally, mangrove leaves of *Avicennia marina (Av. marina)*, and different reef photosymbiotic organisms, including the soft coral *Xenia* sp., the hard coral *Acropora hemprichii (Ac. hemprichii)*, and the jellyfish *Cassiopeia andromeda* (*Cs. andromeda).* The highest FA contents across species were observed in *Leyanella* sp. and *Minutocellus* sp. (diatoms), and *Padina* sp. (macroalgae) strain 1, with means of approximately 17.8, 17.1, and 15.5 mg g^−1^ DW, respectively (Figure 1a). The lowest FA contents across species were found in *Halimeda* sp.(macroalgae), *Trichodesmium* sp. bloom (cyanobacteria), and *Prorocentrum* sp. (dinoflagellate) at 4, 1.8, and 1.7 mg g^−1^ DW (Figure 1a). Diatoms showed notable, yet not significant, variability; *Leyanella* sp. had nearly triple the FA content of *Thalassiosira* sp. (6.4 mg g^−1^ DW; Figure 1a).

Lipid content per DW showed an average value of 3% (range = 1–10%). Lipid content varied significantly across species (*p* < 0.0001; Kruskal–Wallis test; Figure 1b). Inter-specific variations were significant within the groups of cnidarians and macroalgae (*p* < 0.05; Kruskal–Wallis test), but not within cyanobacteria and eukaryotic microalgae (Figure 1a). The highest lipid content across species was found in *Av. marina* (mangrove) leaves (Figure 1b). The lowest lipid contents were observed in *Cs. andromeda* (jellyfish) and *Trichodesmium* sp. bloom (cyanobacteria; Figure 1b). The two strains of *Padina* sp. (macroalgae) showed significant variations in both lipid and FA contents.

### 2.2. Fatty Acid Profiles

**Figure 2:**
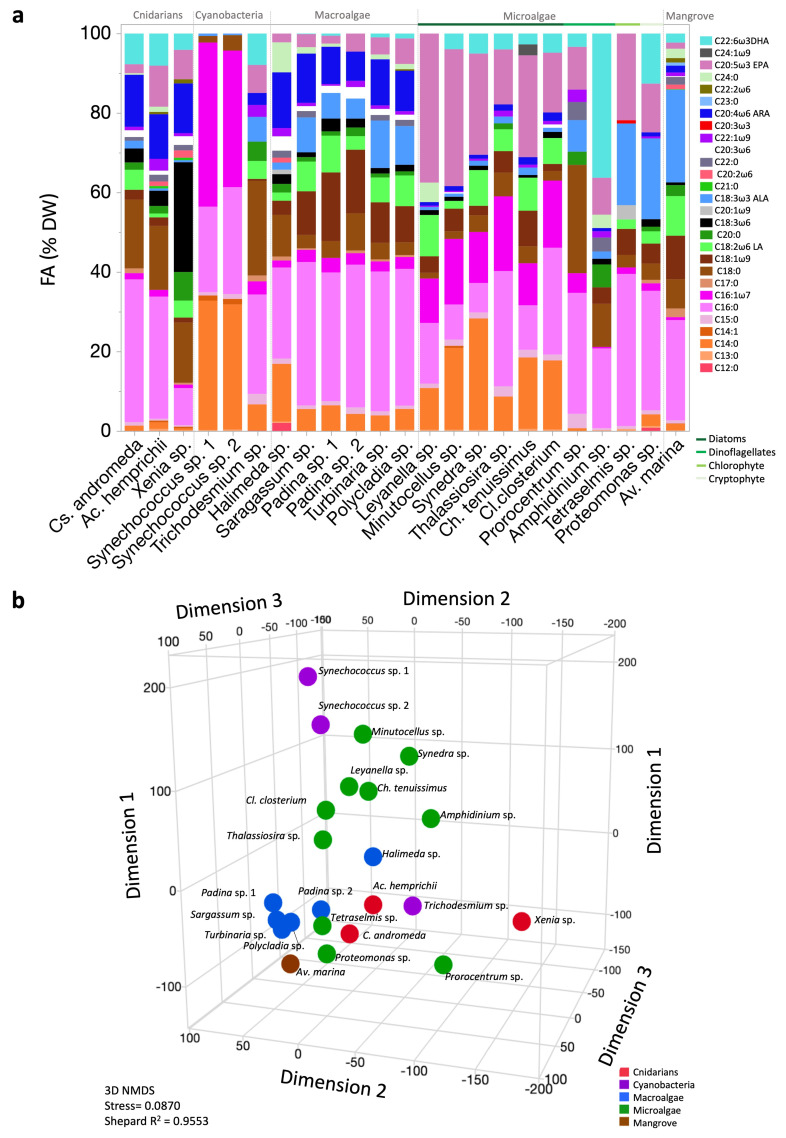
Fatty acid (FA) profiles and patterns of Red Sea photobiota. (**a**) FA profiles (%DW); DW, dry weight. (**b**) Three-dimensional nonmetric multidimensional scaling (NMDS) ordination of mean FA percentage data.

We detected 28 FAs across the studied organisms. The five FAs present in all groups were palmitic acid (C16:0), stearic acid (C18:0), pentadecanoic acid (C15:0), palmitoleic acid (C16:1ω7), and ALA (C18:3ω3; Figure 2a). Comparisons between certain FAs observed in the profiles of both the diatoms and macroalgae (Figure 2a) show that diatoms exhibited relatively higher mean EPA, C14:0, C16:0, C16:1ω7, and DHA contents, with a notably high EPA/DHA ratio compared to macroalgae. In contrast, macroalgae exhibited high mean C16:0, C18:1ω9, ARA, and ALA contents, with significantly low EPA and DHA compared to diatoms. Multivariate analysis indicated the distances among the organisms based on their FA profiles, revealing the presence of both clustering and overlap (stress = 0.087; Figure 2b), showing how some organisms, such as the cyanobacteria *Trichodesmium* sp., separated from the other cyanobacteria analyzed.

### 2.3. Nutritional Quality Assessment

**Figure 3:**
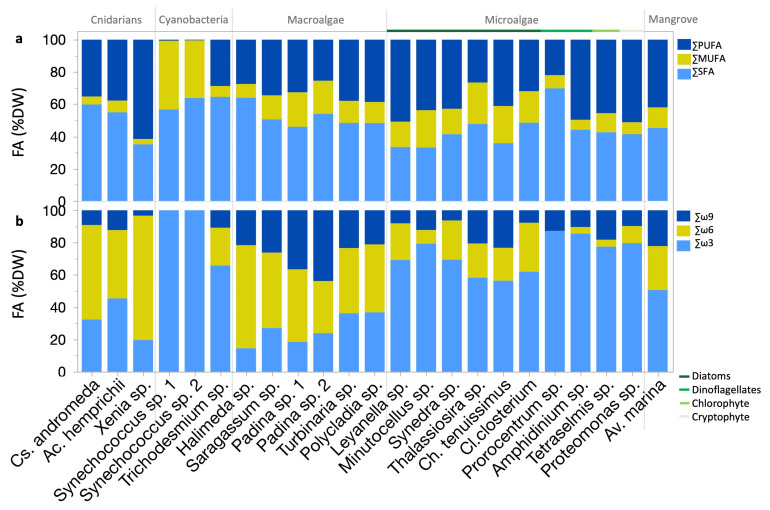
FA nutritional quality metrics for Red Sea photobiota: (**a**) Percentages of saturated FA (SFA), monounsaturated FA (MUFA), and polyunsaturated FA (PUFA) of total FA. (**b**) ω3, ω6, and ω9 relative contents.

In cnidarians, the order of FA contents saturation was SFA > PUFA > MUFA. *Xenia* sp. (soft coral) exhibited higher PUFA contents compared to *C. andromeda* (jellyfish; *p* < 0.01; Dunn test), with a saturation order of PUFA > SFA > MUFA. Conversely, *Cs. andromeda* and *Ac. hemprichii* (hard coral) showed an order of SFA > PUFA > MUFA. (Table S1 and Figure 3a).

Macroalgae generally displayed an order of SFA > PUFA > MUFA. SFA, PUFA, and MUFA contents varied among strains (*p* < 0.05; Dunn test; Table S1 and Figure 3a). The *Av. marina* mangrove leaves exhibited nearly equal PUFA and SFA contents, both higher than the MUFA content.

Eukaryotic microalgae typically exhibited a saturation order of PUFA > SFA > MUFA (Figure 3a). The percentage of SFA content in relation to PUFA and MUFA was significantly higher in *Prorocentrum* sp. (dinoflagellate) compared to other eukaryotic microalgae (Table S1 and Figure 3a).

*Prorocentrum* sp., macroalgae *Padina* sp. strain *2*, *Sargassum* sp., and *Halimeda* sp., cyanobacteria *Trichodesmium* sp. bloom, and *Synechococcus* spp., as well as the zooxanthellae cnidarians *Ac. hemprichii*, and *Cs. andromeda*, all contained over 50% SFA (Table S1 and Figure 3a). The *Proteomonas* sp. (cryptophyte)*, Leyanella* sp. (diatom), and *Xenia* sp. (soft coral) exceeded 50% PUFA (Table S1 and Figure 3a). Generally, MUFA contents were lower than both SFA and PUFA contents, except in *Synechococcus* spp. (cyanobacteria), where MUFAs surpassed SFA content (Table S1 and Figure 3a). PUFA/SFA ratios were higher in eukaryotic microalgae than in both cyanobacteria (*p* < 0.0001; Dunn test) and macroalgae (*p* < 0.01; Dunn test; Figure S1a). PUFA/SFA ratios were highest in *Xenia* sp. and *Leyanella* sp. and showed the lowest contents in *Synechococcus* sp. strain 2 (*p* < 0.05; Dunn test).

ω3, -6, and -9 FAs exhibited mean contents of 2.1, 1.6, and 0.8 mg g^−1^ DW, respectively, with significant group variations (*p* < 0.0001; Kruskal–Wallis test). ω3 was highest in eukaryotic microalgae (i.e., 50–87% of their FA profiles) and lowest in cyanobacteria and macroalgae (*p* < 0.001; Dunn test; Table S1). ω6 contents were highest in cnidarians and macroalgae and were lowest in eukaryotic microalgae and cyanobacteria (*p* < 0.001; Dunn test). ω9 was the most abundant in *Av. marina* mangrove leaves and lowest in cyanobacteria (*p* < 0.001; Dunn test).

Extensive species-specific differences were observed in ω3, −6, and −9 (*p* < 0.0001; Kruskal–Wallis test; Table S1 and Figure 3b). Key ω3 producers were the diatoms *Leyanella* sp., and *Minutocellus* sp., and *Proteomonas* sp. (cryptophyte; Table S1). Elevated ω6 contents were observed in the *Xenia* sp. (soft coral) and *Padina* sp. (macroalga) strain 1 (Table S1). Elevated ω9 contents were observed in *Padina* sp. strain 1 (Table S1). Generally, ω9 is less common than ω3 and ω6, except in *Padina* spp., where it surpassed ω3 (Figure 3b). The *Acropora hemprichii* (hard coral) had almost equal contents of ω3 and ω6, with ω3 exceeding ω6 by 3% (Figure 3b). Conversely, ω6 constituted 64% the FA profile of *Halimeda* sp. (macroalga), a pattern also observed in *Xenia* sp., and *Cs. andromeda* (Figure 3b).

Eukaryotic microalgae exhibited lower ω6/ω3 ratios than cnidarians (*p* < 0.0001; Dunn test; Figure S1b). The highest ω6/ω3 ratios were found in the *Halimeda* sp. (macroalga) and *Xenia* sp. (soft coral), whereas the lowest ratios were observed in the dinoflagellates *Amphidinium* sp. and *Prorocentrum* sp., *Tetraselmis* sp. (chlorophyte), and *Minutocellus* sp. (diatom; *p* < 0.05; Dunn test, Table S1).

The highest atherogenic index (AI) was found in cyanobacteria compared to other groups except macroalgae (*p* < 0.05; Dunn test, Figure S1c). The positive and negative correlations between AI and other indexes of nutritional quality are demonstrated in a correlation matrix (Figure S2).

Principal component analysis (PCA) was conducted based on the FA nutritional quality indexes and five EFAs (Figure S3). The first two components explained 66.9% of the variance, with PC1 accounting for 43.8% and PC2 for 23.1%. The diatom *Leyanella* sp., located outside the 95% confidence ellipse, demonstrates distinct FA nutritional quality.

### 2.4. Polyunsaturated Fatty Acid Content and Composition

**Figure 4:**
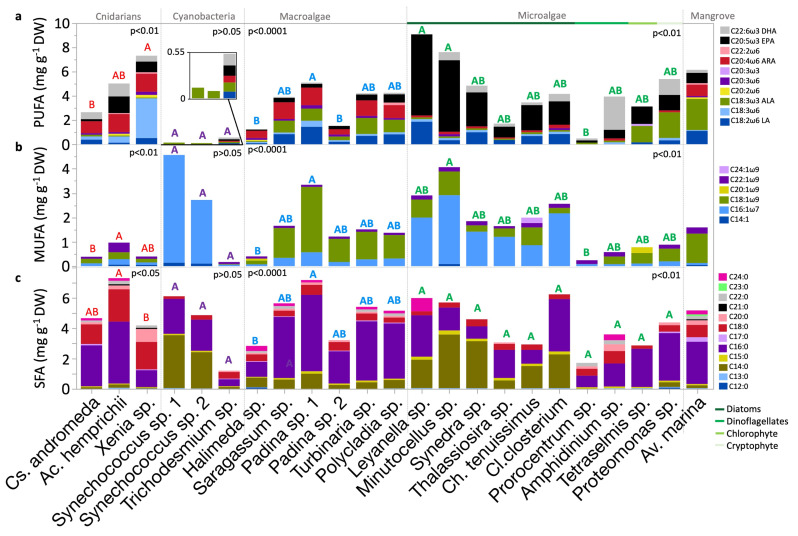
Distribution of FA content according to saturation across Red Sea biota within their taxonomic groups (**a**) polyunsaturated FA (PUFA), (**b**) monounsaturated FA (MUFA), and (**c**) saturated FA (SFA) contents. Bars show means, mg g^−1^ DW. Statistical analyses: Kruskal–Wallis test followed by Dunn test, where species that do not share a letter are significantly different. Color-coded letters correspond to the comparisons within the respective group panel. FA, fatty acid; DW, dry weight.

The identified ω6 PUFAs included C18:3ω6, C20:2ω6, C20:3ω6, C22:2ω6, LA (C18:2ω6), and ARA (C20:4ω6) (Table A1 and Figure 4a). These FAs exhibited both species-specific as well as group-significant variations (*p* < 0.0001, *p* <0.01, or *p* <0.05; Kruskal–Wallis test). No group variations were observed in C20:3ω6 and C22:2ω6. LA contents, averaging 0.6 mg g^−1^ DW, were highest in the diatom *Leyanella* sp. (1.9 mg g^−1^ DW), the macroalga *Padina* sp. strain 1 (1.4 mg g^−1^ DW), and mangrove leaves of *Av. marina* (1.1 mg g^−1^ DW). The specimens of the cyanobacteria *Trichodesmium* sp. sampled during the bloom contained LA, while dinoflagellates *Amphidinium* sp., and *Prorocentrum* sp., and the other cyanobacteria *Synechococcus* spp. did not. C18:3ω6 content was highest in corals and specifically in *Xenia* sp. soft coral (3.3 mg g^−1^ DW). C20:2ω6 was found in few organisms (*p* < 0.01; Kruskal–Wallis test). C22:2ω6 was found in a few organisms (*p* < 0.01; Kruskal–Wallis test) but was highest in *Polycladia* sp. (macroalgae). C20:3ω6 was highest in *Xenia* sp. and *Padina* sp. strain 1 and was absent in cyanobacteria and most eukaryotic microalgae, except for the diatoms *Cl. closterium* and *Minutocellus* sp. **ARA** had an average content of 0.8 mg g^−1^ DW, with the highest contents of approximately 1.5 mg g^−1^ DW observed in the corals *Ac. hemprichii* and *Xenia* sp., followed by *Padina* sp. strain 1 (1.5 mg g^−1^ DW), and *Av. marina* leaves (1 mg g^−1^ DW). Eukaryotic microalgae contained significantly lower ARA contents than cnidarians and macroalgae (*p* < 0.0001, Dunn test). ARA was undetected in *Prorocentrum* sp. (dinoflagellate)*, Tetraselmis* sp. (chlorophyte), and the *Synechococcus* spp. (cyanobacteria).

Identified ω3 PUFAs included C20:3ω3, ALA, EPA, and DHA (Table A1 and Figure 4a). These FAs exhibited high significant variation at both species-specific as well as group levels (*p* < 0.0001; Kruskal–Wallis test). C20:3ω3 was found exclusively in *Tetraselmis* sp. (chlorophyte). ALA contents, averaging 0.6 mg g^−1^ DW, observed highest in *Av. marina* mangrove leaves and *Proteomonas* sp. (cryptophyte; approximately 2 mg g^−1^ DW). ALA was also high in *Tetraselmis* sp. and some macroalgae (approximately 1 mg g^−1^ DW). Significant differences in ALA content existed within cnidarians, with the highest content observed in *Cs. andromeda* compared to the soft coral *Xenia* sp. (*p* < 0.05, Dunn test). Significant ALA variations were also observed within eukaryotic microalgae, significant between *Proteomonas* sp. (cryptophyte) and *Thalssiosira* sp. (diatom) (*p* < 0.05; Dunn test).

The mean EPA content was 1.2 mg g^−1^ DW, exceeding the LA, ALA, and ARA contents. The highest averages were found in eukaryotic microalgae (2.4 mg g^−1^ DW), particularly in *Leyanella* sp. and *Minutocellus* sp. (diatoms). In the *Av. marina* grey mangrove, the EPA content was 0.9 mg g^−1^ DW, comparable to that in some diatoms (e.g., *Thalassiosira* sp.). Notable differences in EPA contents were observed between eukaryotic microalgae and macroalgae, as well as between eukaryotic microalgae and cyanobacteria (*p* < 0.0001 and *p* < 0.01, Dunn test, respectively). In cyanobacteria, EPA was detected only in *Trichodesmium* sp. at 0.1 mg g^−1^ DW and was absent from *Synechococcus* spp. Among diatoms, significant variation in EPA content was observed (*p* < 0.05; Kruskal–Wallis test), between *Leyanella* sp. and *Thalassiosira* sp. (*p* < 0.05; Dunn test).

DHA had the lowest mean among other EFAs (0.6 mg g^−1^ DW). The highest contents were found in eukaryotic microalgae, averaging 0.9 mg g^−1^ DW, particularly in *Amphidinium* sp. dinoflagellate (2.8 mg g^−1^ DW). Conversely, macroalgae exhibited the lowest DHA contents; the highest among them was *Polycladia* sp. (0.1 mg g^−1^ DW). Significant differences in the DHA content were observed between eukaryotic microalgae and macroalgae (*p* < 0.0001; Dunn test). No significant variation was found between the high DHA contents found in *Amphidinium* sp. (dinoflagellate)*, Proteomonas* sp. (cryptophyte)*,* and *Ac. hemprichii* hard coral. Notably, strain-specific variations were observed in the DHA contents of *Padina* sp. macroalgae. DHA was absent from *Leyanella* sp. (diatom)*, Tetraselmis* sp. (chlorophyte), macroalgae *Padina* sp. strain 2, and *Halimeda* sp. In *Av. marina* leaves, DHA content measured 0.3 mg g^−1^ DW, similar to that in *Ch. tenuissimus* (diatom). The highest DHA contents in diatoms reached 0.6 mg g^−1^ DW in *Cl. closterium*, *Synedra* sp., and *Minutocellus* sp.

### 2.5. Monounsaturated Fatty Acid Content and Composition

Six monounsaturated fatty acids (MUFAs) were detected, comprising C14:1, one ω7 FA (C16:1ω7), and four ω9 FAs (C18:1ω9, C20:1ω9, C22:1ω9, and C24:1ω9; Table A1 and Figure 4b). The highest contents of C14:1 were detected in *Synechococcus* spp. (cyanobacteria; ~0.1 mg g^−1^ DW) and in *Minutocellus* sp. (diatom; 0.1 mg g^−1^ DW), making it the sole producer of this MUFA among the studied eukaryotic microalgae. The content of C16:1ω7 was significantly higher in eukaryotic microalgae compared to cnidarians and *Av. marina* mangrove leaves (*p* < 0.01; Dunn test). C16:1ω7 was most abundant in *Synechococcus* spp., with varying contents from 2.6 to 4.4 mg g^−1^ DW. Diatoms also showed high and variable C16:1ω7 contents, ranging from 0.9 mg g^−1^ DW in *Ch. tenuissimus* to 2.8 mg g^−1^ DW in *Minutocellus* sp. (*p* < 0.01; Kruskal–Wallis test). C22:1 content was highest in the *Ac. hemprichii* (hard coral), *Av. marina*, and *Amphidinium* sp. (dinoflagellate). C20:1 was found exclusively in the microalga *Tetraselmis* sp. (0.2 mg g^−1^ DW) and the macroalga *Halimeda* sp. (0.1 mg g^−1^ DW; *p* < 0.05; Kruskal–Wallis test). Nervonic acid (C24:1) was detected only in the diatom *Ch. tenuissimus* at 0.2 mg g^−1^ DW, indicating high specificity among MUFA production within the biota.

### 2.6. Saturated Fatty Acid Content and Composition

Twelve SFAs were identified, from FA C12:0 to C24:0 (Table A1 and Figure 4c). Although *Halimeda* sp. (macroalga) and *Proteomonas* sp. (cryptophyte) exhibited high contents of C12:0 (~0.1 mg g^−1^ DW), yet no significant variation was observed among species and groups. C13:0 was found to be highest in *Leyanella* sp. (diatom) and *Ac. hemprichii* (hard coral). C14:0 was prevalent in cyanobacteria and diatoms. C16:0 was observed highest in macroalgae compared to cyanobacteria and eukaryotic microalgae (*p* < 0.05; Dunn test). C17:0 contents were highest in *Av. marina* (mangrove) at 0.3 mg g^−1^ DW. The highest content of C15:0 was observed in diatoms, particularly *Minutocellus* sp., at 0.3 mg g^−1^ DW. The content of C18:0 was observed to be highest in cnidarians compared to macroalgae, eukaryotic microalgae, and cyanobacteria (*p* < 0.0001; Dunn test). The content of C20:0 was the highest in *Xenia* sp. (soft coral) at 0.9 mg g^−1^ DW, with *Thalassiosira* sp. being the only diatom that produced it. C22:0 was highest in *Amphidinium* sp. (dinoflagellate) and *Ac. hemprichii* (hard coral). C21:0 was produced only by *Av. marina* mangrove leaves and corals, at approximately 0.1 mg g^−1^ DW. C23:0 was exclusive to the *Av. marina* leaves at 0.1 mg g^−1^ DW, whereas C24:0 contents were the highest in *Leyanella* sp. (diatom) followed by *Halimeda* sp. (macroalga).

### 2.7. Comprehensive Intergroup Comparison

**Figure 5:**
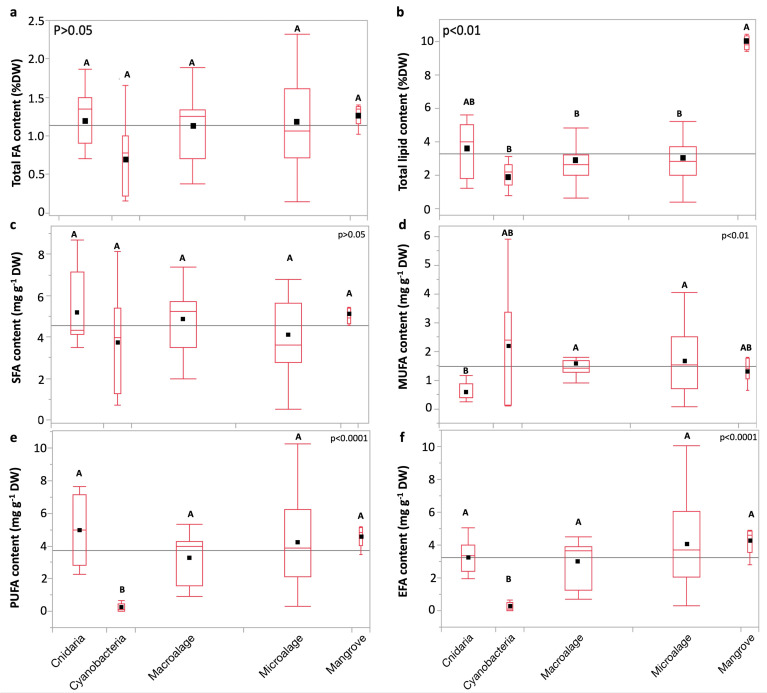
Comparative boxplots of (**a**) total FA, (**b**) total lipid, (**c**) saturated FA (SFA), (**d**) monounsaturated FA (MUFA), (**e**) polyunsaturated FA (PUFA), and (**f**) essential FA (EFA) contents (mean mg g^−1^ DW) across taxa. Replicates from each species and the mean from five biological replicates of leaves of *Av. marina* were used in the comparison. Statistical analyses: Kruskal–Wallis test followed by Dunn test (where the ranks of groups that do not share a letter are significantly different). Black squares represent means. Box edges represent the first and third quartiles (Q1 and Q3), whereas whiskers extend to the minimum and maximum values, and horizontal lines indicate medians. The gray horizontal line represents the grand mean.

Total FA content showed no significant variation between the groups (Figure 5a). Total lipid content varied between the groups (*p* < 0.01; Kruskal–Wallis test; Figure 5d). SFA content varied non-significantly between groups (*p* > 0.05; Dunn test; Figure 5c). The MUFA content was significantly lower in cnidarians than in eukaryotic microalgae, and macroalgae (*p* < 0.01; Dunn test; Figure 5d). PUFA and EFA are lowest in cyanobacteria compared to other groups (*p* < 0.01; Dunn test; Figure 5e,f).

## 3. Discussion

In this research, we analyzed the total lipid contents and FA profiles of 23 organisms from the tropical Red Sea, assessing their nutritional qualities and highlighting their nutraceutical potential quantitatively with a foreknowledge of the variations in their metabolic traits due to their phylogenetic variations [64,65].

More than 75% of previous FA research is based on the relative FA composition as indicated by Colombo et al., which may obscure the nuances that absolute FA content could provide [27]. The quantitative analysis is specifically fundamental for targeted bioprospective studies [66]. Most FA studies of photobiota from the Red Sea are qualitative [48,49,50,51,52,53,54,55,56,57,58], with few absolute quantification studies [45]. Combining a quantitative analysis approach along with relative analysis could potentially provide a comprehensive understanding of FA characteristics [67] and revise currently accepted perspectives on tropical FA quality. For instance, despite the notable variation in relative DHA of both *Amphidinium* sp. (dinoflagellate) and *Ac. hemprichi* (hard coral) 36% and 8% of TFA, respictively, there was no significant variation in their absolute DHA contents. This is relevant for comparing FA profiles of organisms both within the same ecosystem and across different ecosystems, and underscores the need for more quantitative FA research.

In this study, we provide a quantitative FA dataset for *Av. marina* mangrove leaves, addressing a gap in our previous knowledge. In *Av. marina* leaves, ALA, LA, and the >18 carbons SFAs comprised 41% of the FA profile, in agreement with the prevalence of these FAs in vascular plants [14,68]. The presence of EPA and DHA in *Av. marina*, aligns with findings from other studies [14,69,70] and contrasts with others [14,69,70], suggesting the presence of thraustochytrids. Thraustochytrids are PUFA-rich heterotrophic fungus-like micro-organisms known to inhabit leaf surfaces of mangroves and seagrasses [69,71,72,73,74]. An isolated thraustochytrid strain from the Red Sea’s *Av. marina*, identified as *Aurantiochytrium* sp., exhibited a DHA production potential [75]. The natural combination of *Avicennia* leaves and thraustochytrids provides substantial nutrition to organisms such as crabs and camels that consume the leaves [14,76]. Apart from this, *Avicennia* leaves are part of the diet of humans in various cultures [77]. Nevertheless, the FA composition of *Av. marina* has demonstrated antimicrobial and anti-inflammatory properties [78,79], highlighting its extensive benefits and promising applications.

Observing FA profiles can offer insights into the metabolic pathways of an organism. For example, the ability of Red Sea cyanobacteria *Synechococcus* spp. in this study, to synthesize ALA (C18:3ω3) could place the analyzed Red Sea *Synechococcus* spp. in group 3 cyanobacteria possessing the desaturases Δ9,12,15 as classified by Los et al. [80]. However, despite the existence of some *Synechococcus* through literature containing C18- and C20- PUFA, such as EPA [13,80,81], it is generally accepted that the enzymatic capacity of *Synechococcus* is limited to mostly producing SFA and MUFA [82]. The presence of ALA in *Synechococcus* spp. in our study, despite the absence of the known conventional precursors—LA (C18:2ω6) and oleic acid (C18:1ω9) [13,83], will need further studies. Despite the low nutritional quality of *Synechococcus* sp., its ability to synthesize ALA may enhance its nutritional value compared to other counterpart species [84,85]. However, due to lacking several PUFAs of nutritional significance like EPA and DHA, along with its high contents of SFAs, which have lower boiling and oxidation points, *Synechococcus* sp. could be more suitable for applications related to biofuels and pharmaceuticals. On the other hand, the detected EPA and DHA in the cyanobacterium *Trichodesmium* sp. bloom analyzed in this study, aligning with findings from a pure culture of Red Sea *Trichodesmium* sp. [81] and contrasting with *Trichodesmium* sp. elsewhere [86,87] indicates the significant nutritional role of this cyanobacterium in the Red Sea pelagic food web.

We noted variations in the FA composition of the analyzed Red Sea microalgae and their counterparts isolated from other habitats. For instance, the analyzed Red Sea *Synechococcus* spp., demonstrates the metabolic ability to synthesize some FAs (i.e., C13:0, C18:0, C14:1, and ALA), absent in another counterpart species [84]. Another example is Red Sea *Thalassiosira* sp., *Synedra* sp., and *Cl. closterium* synthesized ALA and ARA, absent in their counterparts from elsewhere [88,89,90].

Total lipid content varied among species, but none of them could be classified as oleaginous, as they did not accumulate lipids exceeding 20% of their DW under current growth conditions [91]. The highest and lowest total lipid contents were found in *Av. marina* and *Cs. andromeda*, respectively, with both exceeding some of their tropical and subtropical counterparts [92,93]. The high total lipid contents of *Av. marina* mangrove leaves and *Xenia* sp. soft coral align with previous findings from the Red Sea [57,63]. Additionally, the higher lipid content found in the soft corals compared to the hard coral is consistent with existing literature [57]. Microalgal lipid content can be further enhanced under different growth conditions [94].

Both lipid content and FA in organisms are a function of taxonomy and environmental conditions [94,95]. In our study, the largest differences are associated with taxon variability as the lowest lipid content in jellyfish is mostly due to the high proportion of water (~96%) characterizing this organism [96], while mangrove leaves have high levels of triacylglycerols, sterol esters, and phospholipids [97]. The growth stage influences lipid accumulation, as is reported for microalgae showing higher content by forming more triacylglycerols in the stationary phase [98]. Therefore, all microalgae and cyanobacteria cultures in this study were harvested during the exponential growth phase. Although the growth stage could also affect the FA by either increasing or decreasing certain EFA contents, yet the pattern is variable across organisms, and the growth stage only accounts for approximately 1% of the FA variation, whereas the majority is attributed to taxa [99]. Cultured organisms were grown at Red Sea temperatures and using the hypersaline water of the Red Sea to avoid stressing the cells. Fluctuations in growth conditions, such as the increase in temperature, light, salinity, and nutrients, are known to stress the cells stimulating their lipid accumulation, a cellular strategy for the reuse of stored lipids under favorable conditions [100], as well as influencing their FA contents [45,51,61,101]. Under elevated environmental temperatures, cell membranes adjust by increasing the ratio of SFA to PUFA (including EPA and DHA [25]) to maintain cellular homeostasis [102] through homeoviscous adaptation [103]. Accordingly, tropical marine ecosystems are known for their high proportional (%) SFA, ARA, and ω6 and low PUFA, EPA, DHA, and ω3 contents [27]. In contrast to temperature, salinity and EFA are positively correlated [47,95] as well as the factor of salinity can induce variations in organisms’ FA contents and nutritional quality [3,51]. In addition, the content and changes in lipid accumulation in the zooxanthellate influence the lipid content transferred to the cnidarian host [104], but also the loss of the relationship between corals and their photosymbionts (i.e., coral bleaching) markedly reduces the supply of total lipids, PUFA, and EFAs to the host [105]. Other factors for cnidarians include the host’s spawning season, before which the lipid content is increased, but also food sources can potentially influence the organism’s FA composition [106,107]. The lipids and FA content and composition for the corals in our study corresponded to the fall season separated from the spawning season that occurred during March–April [108]. While we took into consideration controlling some factors influencing lipid and FA contents, yet some factors were not controllable, and variation in the values reported in this study is expected.

In eukaryotic microalgae, we observed the highest nutritional quality. The dinoflagellate *Amphidinium* sp. exhibited the highest DHA content, comprising approximately 36% of its FA profile, which is a known FA indicator for dinoflagellates [13]. High DHA content was also observed in the cryptophyte *Proteomonas* sp., highlighting its potential as a source of DHA. In this study, *Leyanella* sp. and *Minutocellus* sp. exhibited the highest EPA contents among all the organisms analyzed, constituting 34–38% of their total FAs. The absence of previously analyzed FA profiles for *Leyanella* sp. and *Minutocellus* sp. in the existing literature complicates direct comparisons. From a quantitative perspective, the EPA contents of *Leyanella* sp. and *Minutocellus* sp. are comparable to or higher than those reported for other diatoms [84,109]. Modifying growth conditions could further enhance EPA production in these diatoms [110]. *Thalassiosira* sp. compared to other diatoms in this study had the lowest EPA content, however, it is almost similar to the quantity found in counterpart species from elsewhere [111]. Although *Thalassiosira* sp. exhibited low DHA contents among diatoms; yet its DHA content exceeded that of another marine counterpart by 2.5 times, along with its higher total EFAs [111]. Additionally, in the Red Sea *Cl. closterium*, PUFA was predominant over MUFA, and ω3 is higher than ω6 and ω9 in contrast to another *Cl. closterium* study where MUFA exceeded PUFA, and ω9 was the highest [90]. The elevated levels of C16:1ω7 observed in eukaryotic microalgae should be attributed to the large number of diatoms included in the study, as this MUFA is notably enriched in diatom lipids and serves as a precursor in the C16 PUFA pathway, which is predominant in diatoms [112]. In the six analyzed diatoms, we observed high C16:1ω7 to C16:0 and high EPA to DHA ratios, although DHA was totally absent from *Leyanella* sp., consistent with previous knowledge about diatoms [3,13] and distinguishing them from dinoflagellates [113]. The elevated EPA content in diatoms observed in this study aligns with the literature, reaffirming their role as the highest contributors to global EPA production (240 Mt annually) [3,13,114,115]. Nervonic acid (C24:1ω9), a long-chain MUFA found in the white matter of mammalian brains and crucial for neurological development and brain health [116], was exclusively found in *Ch. tenuissimus* (diatom). This finding complements limited reports in the literature on microalgae synthesizing this FA [117].

Our analyzed zooxanthellate cnidarians are known for their symbiotic relationship with dinoflagellates [57,59,118]. The significant DHA content in the hard coral *Ac. hemprichii* might be attributed to its symbiotic dinoflagellate. Although EFAs are traditionally known to be acquired through diet or produced by the photosynthetic symbionts, recent studies have shown that *Acropora* can biosynthesize long-chain EFAs endogenously, contributing to the pool of EFAs [119,120,121,122]. Cnidarians in this study display nutritional qualities more similar to macroalgae, particularly in their elevated ω6/ω3 ratios, but they are nutritionally inferior to eukaryotic microalgae. *Cs. andromeda* exhibited the lowest nutritional quality among cnidarians. The saturation pattern in Red Sea *Acropora*, with SFA > PUFA > MUFA, aligns with patterns observed in other tropical *Acropora* [123]. Conversely, Red Sea *Cs. andromeda* shows a higher SFA to PUFA ratio in contrast to its Mediterranean counterpart [59]. The FAs of the analyzed zooxanthellate cnidarians not only hold pharmaceutical and nutraceutical potential but are also crucial for maintaining the health of the reef ecosystem.

The assessment of nutritional qualities revealed substantial variations among the analyzed macroalgae. We found the highest nutritional values in *Polycladia* sp. and *Turbinaria* sp. (phaeophytes). We observed notable differences in the nutritional qualities between the two *Padina* sp., except for their EPA contents. This could be attributed to several reasons like seasonal or spatial variations [56]. The nutritional quality of *Padina* sp. and *Sargassum* sp. exceeded that of their counterparts elsewhere [124,125,126,127]. By analyzing six macroalgae, some patterns could be observed, such as the FA compositional similarity between the phaeophytes and the chlorophyte, with the former having higher C18:1ω9 than the latter. This observation is in good agreement with the results from a meta-analysis by Kelly and Scheibling [113]. In addition, ARA in this study was predominant in macroalgae [127] compared to microalgae. Among micro- and macroalgae, we observed the MUFA C20:1ω9 to be limited to chlorophytes while C20:2ω6 was found only in *Halimeda* sp. Our study supports previous research indicating the high nutritional values of tropical macroalgae [22,128,129].

The ongoing rapid warming in the Red Sea [43] is expected to decrease the FA nutritional quality of primary producers unless offset by adaptation [25,45,130,131]. However, there is considerable variability in organisms’ responses to temperature and their adaptive capacities, which can contribute to intraspecific variability in FA profiles. When several stressors act simultaneously, they may have antagonistic effects. In other words, the high salinity of the Red Sea (i.e.,~40 [37]) and the organism’s adaptation to this condition by inducing certain unsaturated FAs [47,50,51] could compensate for changes induced by increasing temperature. These predictions highlight the importance of establishing an accurate baseline of the FA profiles of organisms in warming but salty marine ecosystems.

In conclusion, the diverse Red Sea photobiota demonstrated significant nutraceutical potential by synthesizing diverse bioactive FAs despite the extreme conditions of the Red Sea. This highlights the resilience and adaptability of these organisms and underscores our findings in this underexplored ecosystem. The analyzed species are contributors to the quality of human maritime food in the Red Sea; some including the brown seaweeds *Sargassum* sp., *Turbinaria* sp., *Polycladia* sp. (also known as *Cystoseira* sp.), and *Padina* sp. as well as *Tetraselmis* sp. (cryptophyte) are directly edible. The diatoms *Minutocellus* sp. and *Leyanella* sp., with their exceptionally high EPA contents, optimal PUFA/SFA ratios, low ω6/ω3 ratios, and rich lipid profiles, present promising candidates for diverse applications, including nutraceuticals, and aquaculture feed. This potential is contingent upon confirming their FDA “Generally Recognized as Safe” status [132], scaling up production, optimizing extraction methods, and assessing efficacy in target applications. This study highlights the untapped molecular potential of the highly biodiverse Red Sea ecosystem and opens a window for harnessing this biodiversity for bio-based industries [96].

## 4. Materials and Methods

### 4.1. Sample Collection and Identfication

We employed various sample collection, harvesting, and processing methods. The list of studied organisms, sampling location, type of the sample, number of replicates, amount analyzed, and isolation and analysis dates are listed in Table S2. Macroalgae—including *Sargassum* sp., *Turbinaria* sp., *Polycladia* sp. (also known as *Cystoseira* sp.; December 2022), and *Padina* sp. strain 1 (June 2023)—were collected from a coastal station (22.2030 N, 39.0513 E). *Padina* sp. strain 2 and *Halimeda* sp. (April 2022; The Red Sea Decade Expedition) were collected from (22.2724 N, 39.0456 E) and (19.3150 N, 40.0151 E), respectively. The entire thallus was used for macroalgae analysis without specific segmentation or selection of parts. Planktonic diatoms (*Cylindrotheca Closterium*, *Thalassiosira* sp., *Synedra* sp., *Chaetoceros tenuissimus*, *Leyanella* sp., and *Minutocellus* sp.), cryptophyte *Proteomonas* sp. and chlorophyte *Tetraselmis* sp. were isolated in 2015 and dinoflagellates (*Amphidinium* sp. and *Prorocentrum* sp.) in 2016, from open waters or close to Alfahal and Qita Al Kirsh reefs surface water (Table S2). *Synechococcus* sp. strains 1 and 2 were isolated in 2017 from open waters at depths of 0.5 m and 20 m, respectively. All cultures were maintained in axenic stock conditions. However, despite strong axenic conditions, microalgae, including diatoms, are known to harbor cell-attached bacteria [133]. Microalgae and cyanobacteria after successful isolation were maintained in stock cultures growing under Red Sea temperature and salinity conditions in the laboratory, until subcultured and analyzed in August/September 2023. Nitrogen-fixing cyanobacteria *Trichodesmium sp.* were initially sampled in late May 2022 from a large bloom at a pelagic coastal station (22.2030 N, 39.0513 E) using a 15 µm plankton net and subsequently size-fractionated through a 200 µm mesh before final filtration. Photosymbiotic cnidarians, including the soft coral *Xenia* sp. and hard coral *Ac. hemprichii*, were isolated (2021) and maintained in the aquaculture tanks of KAUST Coastal and Marine Resources Core Lab until analyzed in September 2023. The upside-down jellyfish *Cs. andromeda* (June 2022; identified by Aljbour and Agusti [134]) and mangrove leaves of *Av. marina* (July 2022) were collected from coastal areas in Thuwal. The organisms were collected non-simultaneously from different locations due to the diverse nature of the organisms studied, as some are benthic, others are pelagic, some require scuba diving, and others the use of nets. All samples were sourced from the Central Red Sea, Saudi Arabia. Certain natural samples, such as the zooxanthellae-epibenthic jellyfish and *Trichodesmium* bloom samples, which are not available year-round or not in sufficient analytical quantities, were collected during specific occurrences. Identification was conducted using morphological and/or genomic techniques.

Microalgae species were classified by both morphological [40,135] and/or genomics by DNA extraction and 18S rDNA sequencing reads. For identification using genomics, 50 mL of each culture was filtered through a polycarbonate filter, and DNA was extracted using the DNeasy^Ⓡ^ PowerWater^Ⓡ^ DNA Extraction kit (MoBio Laboratories, Inc., Carlsbad, CA, USA). The DNA obtained was quantified with a Qubit^Ⓡ^ fluorimeter (Life Technologies, Carlsbad, CA, USA). PCR amplification was performed using an Eppendorf Mastercycler^Ⓡ^ Pro and the universal LSU rDNA D1-D2 primers [136]. 25 ng of DNA from each culture, Qiagen multiplex PCR master mix (QIAGEN, Valencia, CA, USA), and a final primer concentration of 0.3 µM were used, to a final volume of 30 µL. PCR conditions consisted of an initial activation step at 94 °C for 15 min, 25 cycles of 30 s at 94 °C for denaturation and 30 s at 55 °C for the annealing temperature, followed by an extension step of 45 s at 72 °C, and a final elongation step of 5 min at 72 °C. The obtained PCR products were cleaned using AMPure XP beads (Beckman Coulter, Brea, CA, USA) and checked and quantified using Agilent TapeStation 4200 (Agilent Technologies, Santa Clara, CA, USA). Subsequently, the amplicons were sequenced by Sanger in both directions in the Bioscience Core Laboratory at KAUST. The nucleotide sequences were analyzed using the Basic Local Alignment Search Tool (BLAST) www.ncbi.nlm.nih.gov/BLAST (accessed on 24 October 2024). Species tree based on maximum likelihood were compared with GenBank database sequences using the BLAST tree view at https://www.ncbi.nlm.nih.gov/blast/treeview/treeView.cgi (accessed on 24 October 2024). Species identifications were confirmed when BLAST query covered >99% and showed 100% morphological matching; otherwise, classified at the genus level.

### 4.2. Harvesting and Processing

Eukaryotic microalgae and *Synechococcus* spp. strains were cultured in glass flasks with vented caps at a final volume of 250 mL. We used 0.22-µm-filtered, and autoclaved Red Sea water (Cycle #8, Systec autoclave DE-45, Linden, Germany) to prepare the culture media to maintain Red Sea salinity. The media was enriched with Guillard’s F/2 solution [137] and vitamin B12. Sodium metasilicate nonahydrate (Na_2_ SiO_3_.9H_2_O) was added to the diatom culture (1mL/L). Microalgae cultures were grown at 22 °C (24 °C for *Synechococcus* spp.), temperatures close to the mean annual temperature of the Red Sea (i.e., 26 °C [43]). Culture was grown under light conditions of 12:12 light/dark cycle with white LED irradiance of 51 µmol photons m^−2^ s^−1^ (Percival^®^, IntellusUltra Chamber Control System, USA). Cells were harvested during the exponential growth phase through centrifugation at 7000× *g* for 15 min at 22 °C (JA-14 rotor, Avanti J-26 XP Centrifuge, Beckman Coulter, Krefeld, Germany). *Synechococcus* sp., *Trichodesmium* sp. bloom, and *Prorocentrum* sp. cells were collected on preweighed and precombusted (450 °C for 4 h) 47 mm GF/F filters (nominal pore size 0.7 µm; Whatman; GE Healthcare Life Science, Little Chalfont, UK). *Acropora hemprichii* tissue was separated from its skeleton using filtered, autoclaved seawater and a pressurized N_2_ airbrush. For the leaves of *Av. marina*, we used green fresh leaves. Jellyfish were immediately transferred to the lab in a large icebox, dissected, and flash-frozen in liquid nitrogen within 1 h of sampling. All samples were lyophilized at −55 °C for 24–48 h at 2100 Pa pressure (freeze-dryer Alpha 1-2 LDplus, CHRIST, Osterode am Harz, Germany), then gently homogenized or sliced. The lyophilized samples were stored in glass containers at −80 °C until extraction, conducted within 5 d to a maximum of 9 months.

### 4.3. Extraction of Total Lipids and FAs Methyl Esters

FAs were extracted from lyophilized samples and transformed into FAMEs through direct one-step transesterification, following the Lewis et al. [138] method with modifications. Briefly, the samples were spiked with 25 µL of C19:0 internal standard prepared in n-hexane (1500 µg ml^−1^), and total FA was extracted using methanol, chloroform, and HCl mixture (10:1:1, *v*:*v*:*v*) with sonication. The purpose of using chloroform is to extract lipids, and HCl, which acts as a catalyst is to accelerate the transesterification process. Methanol serves as the methyl donor, reacting with FAs to produce FAMEs. However, unlike base catalysis, acid catalysis requires heat and more time incubation; therefore, we incubated the samples in a water bath with continuous automatic shaking at 85 °C for 2.5 h (WNB 7 -45, Memmert, Germany). Distilled water (1 mL) was added to separate the chloroform lipid-containing phase from the aqueous phase containing proteins and carbohydrates, causing the polar and nonpolar lipids to rise to the top. This lipid phase was purified and extracted twice with 2 mL of hexane–chloroform mixture (4:1, *v*:*v*), vortexed for 15 s, centrifuged (5 min at 3000 rpm, 20 °C), and pooled into a preweighed amber vial. The solvents were evaporated under a stream of nitrogen within approximately 45 min (DB 100/3, Dri-Block^®^, Techne, London, UK). The total extractable lipid content was gravimetrically calculated post-evaporation. After concentrating, FAME extracts were reconstituted in 0.5 mL n-hexane, for a final C19:0 internal standard concentration of 75 µg/mL, for GC/MS analysis.

### 4.4. Analysis and Quantification of FAME

Analysis of FAME was done using gas chromatography (7890B) equipped with a Triple-Quadrupole MS detector (7010B; Agilent Technologies, Santa Clara, CA, USA) in scanning mode and a CP-Sil 88 column (100 m × 250 µm, 0.20 μm film; Agilent, CA, USA). A 1-μL sample was auto-injected (ALEX autosampler, GERSTEL, Mülheim an der Ruhr, Germany) with a 20:1 split ratio at a 280 °C inlet temperature and a 3 mL/min flow rate. Helium served as the carrier gas at a flow rate of 1 mL/min. The column temperature program began at 80 °C, increased to 210 °C at 4 °C/min for 5 min, and then rose to 230 °C for 19.5 min. Electron impact mass spectra were recorded at 70 eV, covering a range of 35–700 *m/z*, with the MS source set at 230 °C. The total run time was 62 min. Methyl esters were identified by comparing their retention times between sample extracts and those of the FAME standard mix (Supelco^®^ 37 Component FAME Mix, Sigmaaldrich, St. Louis, MO, USA). Quantification of chromatogram peaks was performed using a 7-point calibration curve for each FA for absolute FA quantification [139,140]. The calibration curve solution is prepared from the external standard 37-FAME mix (10–170 µg ml^−1^) in n-hexane and the internal standard FAME C19:0 of 75 µg/mL (500 µg ml^−1^ in n-hexane). These calibrations were analyzed with the samples under the same GC/MS conditions.

**Figure 6:**
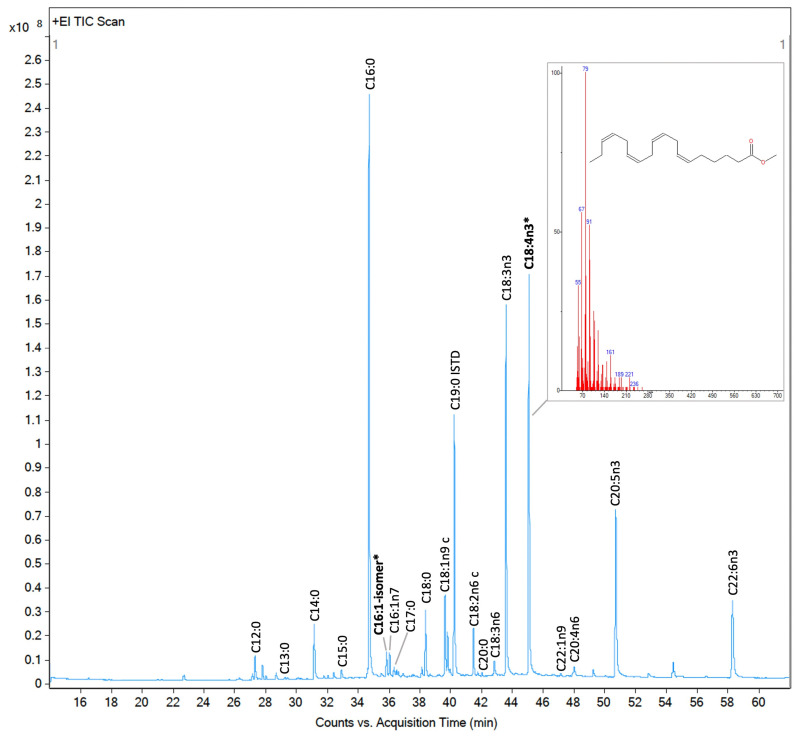
Total ion current chromatogram of fatty acid methyl esters (FAMEs) detected by GC/MS for *Proteomonas* sp. (cryptophyte). FAMEs were annotated and quantified using the 37-component FAME mix and a calibration curve prepared from it. FAMEs marked in bold and with an asterisk were identified based on their mass spectra using the NIST library.

The number of chromatogram peaks of the analyzed photobiota exceeded the quantified FA peaks, with a total of approximately 40 peaks varying between organisms (Figure 6). However, not all peaks observed in the chromatogram corresponded to FAs, as we observed from analyzing the mass spectra of randomly selected peaks using the NIST MS Search 2.2; the chromatogram also contained all extractable derivatized lipids, including non-FAs. The quantified peaks were annotated based on comparisons of the retention times of the samples with those of external standards, specifically the 37-FAME mix, analyzed in GC/MS with the samples under the same conditions.

### 4.5. Nutraceutical and Pharmaceutical Indexes

All quantified FAs were grouped according to their saturation level and omega family to calculate the quality indexes [4,6,141,142].

SFA=(C12:0)+(C13:0)+(C14:0)+(C15:0)+(C16:0)+(C17:0)+(C18:0)         +(C20:0)+(C21:0)+(C22:0)+(C23:0)+(C24:0)

MUFA=(C14:1)+(C16:1ω7)+(C18:1ω9)+(C20:1ω9 cis)+(C22:1ω9)+(C24:1ω9)

PUFA=(C18:2ω6)+(C18:3ω3)+(C18:3ω6)+(C20:4ω6)+(C20:2ω6)                                    +(C20:3ω6)+(C20:3ω3)+(C22:2ω6)+(C20:5ω3)+(C22:6ω3)

$$\omega 6/\omega 3=((C18:2\omega 6\;)+(C18:3\omega 6)+(C20:2\omega 6)+(C20:4\omega 6)\;\;\;\;\;\;\;\;\;\;\;\;\;\;\;\;\;\;\;\;\;\;\;\;\;\;\;\;\;\;\;\;\;\;\;\;\;\;\;\;\;\;\;\;\;\;\;\;\;\;\;\;\;\;\;\;\;\;\;\;\;\;\;\;\;\;\;\;\;\;\;+(C22:2\omega 6))/((C18:3\omega 3)+(C20:3\omega 3)+(C20:5\omega 3)+\left(C22:6\omega 3\right))$$

ω9=(C18:1ω9)+(C20:1ω9)+(C22:1ω9)+(C24:1ω9)

AI=((C12:0)+4(C14:0)+(C16:0))/((∑MUFA+∑PUFA))

EFAs=(C18:2ω6)+(C18:3ω3)+(C20:4ω6)+(C20:5ω3)+(C22:6ω3)

where n in Cn:mωx, is the number of carbon atoms, m is the number of double bonds, and *x* is the number of atoms at which the double bonds are placed from the methyl (omega) end. The FA concentrations (mg g^−1^ DW) were used to derive the total index value.

### 4.6. Software and Statistical Analysis

FA chromatogram peaks were annotated and quantified using Mass-hunter software (Agilent). FA content quantification was based on peaks annotated using the 37 FAME mix external standard, primarily through retention times and calibration curves. The sum of quantified FAME is referred to as total FA content after normalized to the analyzed sample’s dry weight. The NIST library provided confirmation and facilitated the identification of additional peaks via mass spectra. Data are presented as mean ± SE. Normality and homogeneity of variance were assessed using Shapiro–Wilk and Levene’s tests, respectively. Significant differences were determined by using nonparametric Kruskal–Wallis test (rank sums) followed by Dunn’s test for all-pairs joint ranking comparisons, with a significance level set at *p* ≤ 0.05. Data analyses, including Pearson correlation, 3D NMDS, and PCA, were conducted using JMP Pro 16.0 and 18.0. The previous analyses were based on five replicates for macroalgae and cnidarian species and three for eukaryotic microalgae and cyanobacteria, except for *Synechococcus* sp. strain 1, which was analyzed in duplicate. All FA contents were normalized to DW and then expressed as mg g^−1^ DW or transformed to %DW. Graphical abstract created in BioRender.

## Data Availability

The original contributions presented in this study are included in the article/Supplementary Material. Further inquiries can be directed to the corresponding author. Microalgae sequences are deposited in Genbank under the accession numbers *Chaetoceros tenuissimus*, PQ480815; *Thalassiosira* sp., PQ480816; *Cylindrotheca Closterium*, PQ480817; *Leyanella* sp., PQ480818; *Synedra* sp., PQ480820; *Minutocellus* sp., PQ480821; *Tetraselmis* sp., PQ480822; and *Proteomonas* sp., PQ480823.

## Supplementary Materials

The following supporting information can be downloaded at: https://www.mdpi.com/article/10.3390/molecules30030621/s1, Figure S1. Comparative boxplot of FA quality indexes illustrating Red Sea photobiota group variations; Figure S2. Correlation matrix of FA quality indexes across Red Sea photobiota groups; Figure S3. Principal component analysis of Red Sea photobiota based on their FA nutritional quality. Figure S4: Distribution of FA content according to saturation across species; Table S1: FA quality indexes of Red Sea organisms; Table S2: List of studied organisms, sampling location, type of sample, number of replicates, amount analyzed, and isolation and analysis dates.

## Appendix A. Table A1: FA Content and Profiles of Red Sea Organisms

**Table A1:** FA content and profiles of Red Sea organisms (mean ± SE), mg g^−1^ DW; n.d. means “not detected”. Sections divide the SFA, MUFA, and PUFA groups. PUFA, polyunsaturated FA; MUFA, monounsaturated FA; SFA, saturated FA; DW, dry weight.

	***Ac. hemprichii***	***Cs. andromeda***	***Xenia* sp.**	***Av. marina***	***Halimeda* sp.**	***Padina* sp. *1***	***Padina* sp. *2***		***Saragassum* sp.**	***Turbinaria* sp.**	***Polycladia* sp.**	***Amphidinium* sp.**
C12:0	0.003 ± 0.0	0.003 ± 0.0	n.d.	0.008 ± 0.0	0.087 ± 0.0	n.d.	0.001 ± 0.0		n.d.	n.d.	n.d.	n.d.
C13:0	0.066 ± 0.0	n.d.	0.019 ± 0.0	0.039 ± 0.0	0.024 ± 0.0	n.d.	n.d.		n.d.	n.d.	0.030 ± 0.0	0.040 ± 0.0
C14:0	0.217 ± 0.0	0.099 ± 0.0	0.054 ± 0.0	0.186 ± 0.0	0.626 ± 0.1	0.999 ± 0.0	0.254 ± 0.0		0.611 ± 0.0	0.432 ± 0.0	0.554 ± 0.0	n.d.
C15:0	0.062 ± 0.0	0.066 ± 0.0	0.046 ± 0.0	0.087 ± 0.0	0.057 ± 0.0	0.163 ± 0.0	0.097 ± 0.0		0.102 ± 0.0	0.116 ± 0.0	0.088 ± 0.0	0.113 ± 0.0
C16:0	4.081 ± 0.4	2.695 ± 0.3	1.112 ± 0.0	2.780 ± 0.1	0.988 ± 0.1	5.034 ± 0.0	2.130 ± 0.2		4.022 ± 0.1	3.900 ± 0.1	3.637 ± 0.1	1.522 ± 0.2
C17:0	n.d.	0.090 ± 0.0	0.055 ± 0.0	0.301 ± 0.0	0.043 ± 0.0	n.d.	0.040 ± 0.0		0.043 ± 0.0	0.057 ± 0.0	0.053 ± 0.0	n.d.
C18:0	2.138 ± 0.3	1.298 ± 0.1	1.802 ± 0.0	0.811 ± 0.1	0.452 ± 0.1	0.659 ± 0.1	0.555 ± 0.1		0.375 ± 0.1	0.462 ± 0.1	0.338 ± 0.0	0.823 ± 0.2
C20:0	0.252 ± 0.0	0.139 ± 0.0	0.855 ± 0.0	0.305 ± 0.0	0.094 ± 0.0	0.167 ± 0.0	0.129 ± 0.0		0.126 ± 0.0	0.111 ± 0.0	0.109 ± 0.0	0.436 ± 0.1
C21:0	0.080 ± 0.0	n.d.	0.073 ± 0.0	0.068 ± 0.0	n.d.	n.d.	n.d.		n.d.	n.d.	n.d.	n.d.
C22:0	0.227 ± 0.0	0.116 ± 0.0	0.163 ± 0.0	0.213 ± 0.0	0.127 ± 0.0	n.d.	n.d.		0.178 ± 0.0	0.156 ± 0.0	0.164 ± 0.0	0.267 ± 0.0
C23:0	n.d.	n.d.	n.d.	0.110 ± 0.0	n.d.	n.d.	n.d.		n.d.	n.d.	n.d.	n.d.
C24:0	0.174 ± 0.0	0.150 ± 0.0	n.d.	0.260 ± 0.0	0.325 ± 0.1	0.154 ± 0.0	n.d.		0.181 ± 0.0	0.169 ± 0.0	0.175 ± 0.0	0.382 ± 0.0
C14:1	0.061 ± 0.0	n.d.	0.052 ± 0.0	0.048 ± 0.0	n.d.	n.d.	n.d.		n.d.	n.d.	n.d.	n.d.
C16:1ω7	0.224 ± 0.0	0.117 ± 0.0	0.101 ± 0.0	0.080 ± 0.0	0.076 ± 0.0	0.569 ± 0.0	0.171 ± 0.0		0.341 ± 0.0	0.280 ± 0.0	0.315 ± 0.0	0.083 ± 0.0
C18:1ω9	0.286 ± 0.0	0.187 ± 0.0	0.149 ± 0.0	1.206 ± 0.3	0.153 ± 0.0	2.684 ± 0.1	0.950 ± 0.1		1.224 ± 0.0	1.130 ± 0.0	0.962 ± 0.0	0.314 ± 0.1
C20:1ω9	n.d.	n.d.	n.d.	n.d.	0.082 ± 0.0	n.d.	n.d.		n.d.	n.d.	n.d.	n.d.
C22:1ω9	0.395 ± 0.0	0.082 ± 0.0	0.093 ± 0.0	0.255 ± 0.0	0.090 ± 0.0	0.087 ± 0.0	0.093 ± 0.0		0.091 ± 0.0	0.099 ± 0.0	0.092 ± 0.0	0.180 ± 0.0
C24:1ω9	n.d.	n.d.	n.d.	n.d.	n.d.	n.d.	n.d.		n.d.	n.d.	n.d.	n.d.
C18:2ω6	0.129 ± 0.0	0.378 ± 0.0	0.508 ± 0.0	1.099 ± 0.3	0.089 ± 0.0	1.437 ± 0.0	0.202 ± 0.0		0.833 ± 0.0	0.692 ± 0.0	0.816 ± 0.0	n.d.
C18:3ω6	0.510 ± 0.1	0.264 ± 0.0	3.282 ± 0.1	0.073 ± 0.1	0.106 ± 0.0	0.501 ± 0.0	0.133 ± 0.0		0.133 ± 0.0	0.154 ± 0.0	0.175 ± 0.0	0.163 ± 0.0
C20:2ω6	0.151 ± 0.0	n.d.	0.220 ± 0.0	0.127 ± 0.0	0.088 ± 0.0	n.d.	n.d.		n.d.	n.d.	n.d.	n.d.
C20:3ω6	0.136 ± 0.0	0.128 ± 0.0	0.261 ± 0.0	0.107 ± 0.0	0.155 ± 0.0	0.279 ± 0.0	0.172 ± 0.0		0.138 ± 0.0	0.169 ± 0.0	0.138 ± 0.0	n.d.
C20:4ω6	1.491 ± 0.1	0.982 ± 0.1	1.486 ± 0.0	0.941 ± 0.0	0.604 ± 0.1	1.457 ± 0.0	0.436 ± 0.0		1.383 ± 0.0	1.285 ± 0.0	1.075 ± 0.0	0.183 ± 0.0
C22:2ω6	0.077 ± 0.0	n.d.	0.125 ± 0.0	0.123 ± 0.0	n.d.	n.d.	n.d.		n.d.	n.d.	0.207 ± 0.0	n.d.
C18:3ω3	0.077 ± 0.0	0.146 ± 0.0	0.063 ± 0.0	2.572 ± 0.6	0.077 ± 0.0	1.003 ± 0.0	0.299 ± 0.0		0.980 ± 0.0	1.323 ± 0.0	1.030 ± 0.0	0.138 ± 0.0
C20:3ω3	n.d.	n.d.	n.d.	n.d.	n.d.	n.d.	n.d.		n.d.	n.d.	n.d.	n.d.
C20:5ω3	1.371 ± 0.1	0.170 ± 0.0	0.880 ± 0.0	0.850 ± 0.0	0.095 ± 0.0	0.309 ± 0.0	0.269 ± 0.0		0.364 ± 0.0	0.478 ± 0.0	0.679 ± 0.0	0.704 ± 0.2
C22:6ω3	1.074 ± 0.1	0.578 ± 0.1	0.486 ± 0.0	0.250 ± 0.2	n.d.	0.104 ± 0.0	n.d.		0.081 ± 0.0	0.105 ± 0.0	0.130 ± 0.0	2.750 ± 0.8
**FA**	***Prorocentrum* sp. **	***Ch. tenuissimus***	***Cl.closterium***	***Leyanella* sp.**	***Minutocellus* sp.**	***Synedra* sp.**	***Thalassiosira* sp.**	***Proteomonas* sp.**	***Tetraselmis* sp.**	***Synechococcus* sp. 1**	***Synechococcus* sp. 2**	***Trichodesmium* sp.**
C12:0	n.d.	n.d.	n.d.	n.d.	n.d.	n.d.	n.d.	0.081 ± 0.0	n.d.	n.d.	n.d.	0.002 ± 0.0
C13:0	n.d.	0.043 ± 0.0	0.047 ± 0.0	0.051 ± 0.0	0.048 ± 0.0	0.042 ± 0.0	n.d.	0.045 ± 0.0	0.046 ± 0.0	0.049 ± 0.0	0.026 ± 0.0	n.d.
C14:0	0.017 ± 0.0	1.462 ± 0.2	2.229 ± 0.1	1.874 ± 0.2	3.530 ± 0.1	3.092 ± 1.0	0.553 ± 0.1	0.309 ± 0.0	n.d.	3.486 ± 1.2	2.394 ± 0.2	0.121 ± 0.0
C15:0	0.091 ± 0.0	0.156 ± 0.0	0.193 ± 0.0	0.200 ± 0.0	0.270 ± 0.0	0.166 ± 0.0	0.164 ± 0.0	0.107 ± 0.0	0.076 ± 0.0	0.088 ± 0.0	0.093 ± 0.0	0.048 ± 0.0
C16:0	0.761 ± 0.0	0.913 ± 0.2	3.448 ± 0.3	2.726 ± 0.4	1.513 ± 0.1	0.817 ± 0.3	1.857 ± 0.2	3.132 ± 0.4	2.524 ± 0.2	2.306 ± 0.7	2.047 ± 0.2	0.460 ± 0.1
C17:0	n.d.	n.d.	n.d.	n.d.	n.d.	n.d.	n.d.	0.092 ± 0.0	n.d.	n.d.	n.d.	0.041 ± 0.0
C18:0	0.453 ± 0.2	0.347 ± 0.1	0.314 ± 0.0	0.260 ± 0.0	0.333 ± 0.0	0.466 ± 0.3	0.384 ± 0.1	0.427 ± 0.0	0.208 ± 0.0	0.176 ± 0.0	0.296 ± 0.1	0.438 ± 0.2
C20:0	0.166 ± 0.0	n.d.	n.d.	n.d.	n.d.	n.d.	0.096 ± 0.0	0.182 ± 0.0	n.d.	n.d.	n.d.	0.090 ± 0.0
C21:0	n.d.	n.d.	n.d.	n.d.	n.d.	n.d.	n.d.	n.d.	n.d.	n.d.	n.d.	n.d.
C22:0	0.228 ± 0.0	n.d.	n.d.	n.d.	n.d.	n.d.	n.d.	n.d.	n.d.	n.d.	n.d.	n.d.
C23:0	n.d.	n.d.	n.d.	n.d.	n.d.	n.d.	n.d.	n.d.	n.d.	n.d.	n.d.	n.d.
C24:0	n.d.	n.d.	n.d.	0.875 ± 0.1	n.d.	n.d.	n.d.	n.d.	n.d.	n.d.	n.d.	n.d.
C14:1	n.d.	n.d.	n.d.	n.d.	0.088 ± 0.0	n.d.	n.d.	n.d.	n.d.	0.140 ± 0.0	0.106 ± 0.0	n.d.
C16:1ω7	0.083 ± 0.0	0.860 ± 0.1	2.169 ± 0.1	1.994 ± 0.2	2.825 ± 0.1	1.414 ± 0.5	1.203 ± 0.1	0.196 ± 0.0	0.107 ± 0.0	4.423 ± 1.3	2.612 ± 0.3	0.061 ± 0.0
C18:1ω9	0.004 ± 0.0	0.729 ± 0.1	0.223 ± 0.0	0.740 ± 0.1	0.971 ± 0.0	0.260 ± 0.0	0.345 ± 0.0	0.522 ± 0.1	0.432 ± 0.0	n.d.	n.d.	0.027 ± 0.0
C20:1ω9	n.d.	n.d.	n.d.	n.d.	n.d.	n.d.	n.d.	n.d.	0.237 ± 0.0	n.d.	n.d.	n.d.
C22:1ω9	0.156 ± 0.0	0.178 ± 0.0	0.167 ± 0.0	0.168 ± 0.0	0.178 ± 0.0	0.170 ± 0.0	0.095 ± 0.0	0.163 ± 0.0	n.d.	n.d.	n.d.	0.082 ± 0.0
C24:1ω9	n.d.	0.224 ± 0.0	n.d.	n.d.	n.d.	n.d.	n.d.	n.d.	n.d.	n.d.	n.d.	n.d.
C18:2ω6	n.d.	0.677 ± 0.1	0.839 ± 0.0	1.855 ± 0.2	0.335 ± 0.0	0.991 ± 0.3	0.351 ± 0.1	0.322 ± 0.0	0.158 ± 0.0	n.d.	n.d.	0.083 ± 0.0
C18:3ω6	n.d.	0.161 ± 0.0	0.196 ± 0.0	0.220 ± 0.0	0.178 ± 0.0	0.183 ± 0.0	n.d.	0.197 ± 0.0	n.d.	n.d.	n.d.	n.d.
C20:2ω6	n.d.	n.d.	n.d.	n.d.	n.d.	n.d.	n.d.	n.d.	n.d.	n.d.	n.d.	n.d.
C20:3ω6	n.d.	n.d.	0.164 ± 0.0	n.d.	0.184 ± 0.0	n.d.	n.d.	n.d.	n.d.	n.d.	n.d.	n.d.
C20:4ω6	n.d.	0.155 ± 0.0	0.276 ± 0.0	0.161 ± 0.0	0.200 ± 0.0	0.163 ± 0.0	0.101 ± 0.0	0.158 ± 0.0	n.d.	n.d.	n.d.	0.084 ± 0.0
C22:2ω6	n.d.	n.d.	n.d.	n.d.	n.d.	n.d.	n.d.	n.d.	n.d.	n.d.	n.d.	n.d.
C18:3ω3	0.133 ± 0.0	0.153 ± 0.0	0.134 ± 0.0	0.143 ± 0.0	0.137 ± 0.0	0.138 ± 0.0	0.109 ± 0.0	2.117 ± 0.2	1.355 ± 0.1	0.133 ± 0.0	0.091 ± 0.0	0.115 ± 0.0
C20:3ω3	n.d.	n.d.	n.d.	n.d.	n.d.	n.d.	n.d.	n.d.	0.164 ± 0.0	n.d.	n.d.	n.d.
C20:5ω3	0.179 ± 0.0	2.086 ± 0.3	1.930 ± 0.1	6.695 ± 0.8	5.904 ± 0.0	2.808 ± 1.1	0.887 ± 0.1	1.278 ± 0.2	1.439 ± 0.1	n.d.	n.d.	0.130 ± 0.0
C22:6ω3	0.167 ± 0.0	0.222 ± 0.0	0.618 ± 0.0	n.d.	0.673 ± 0.0	0.554 ± 0.2	0.255 ± 0.0	1.312 ± 0.2	n.d.	n.d.	n.d.	0.145 ± 0.0

## References

[1] Twining C.W., Bernhardt J.R., Derry A.M., Hudson C.M., Ishikawa A., Kabeya N., Kainz M.J., Kitano J., Kowarik C., Ladd S.N. (2021). The evolutionary ecology of fatty-acid variation: Implications for consumer adaptation and diversification. Ecol. Lett..

[2] Galloway A.W.E., Budge S.M. (2020). The critical importance of experimentation in biomarker-based trophic ecology. Philos. Trans. R. Soc. B Biol. Sci..

[3] Cañavate J.P. (2019). Advancing assessment of marine phytoplankton community structure and nutritional value from fatty acid profiles of cultured microalgae. Rev. Aquac..

[4] Chen J., Liu H. (2020). Nutritional Indices for Assessing Fatty Acids: A Mini-Review. Int. J. Mol. Sci..

[5] Rincon-Cervera M.A., Gonzalez-Barriga V., Romero J., Rojas R., Lopez-Arana S. (2020). Quantification and Distribution of Omega-3 Fatty Acids in South Pacific Fish and Shellfish Species. Foods.

[6] Zisis F., Kyriakaki P., Satolias F.F., Mavrommatis A., Simitzis P.E., Pappas A.C., Surai P.F., Tsiplakou E. (2022). The Effect of Dietary Inclusion of Microalgae Schizochytrium spp. on Ewes’ Milk Quality and Oxidative Status. Foods.

[7] Waehler R. (2021). Fatty acids: Facts vs. fiction. Int. J. Vitam. Nutr. Res..

[8] Khan I., Hussain M., Jiang B., Zheng L., Pan Y., Hu J., Khan A., Ashraf A., Zou X. (2023). Omega-3 long-chain polyunsaturated fatty acids: Metabolism and health implications. Prog. Lipid Res..

[9] Djuricic I., Calder P.C. (2021). Beneficial Outcomes of Omega-6 and Omega-3 Polyunsaturated Fatty Acids on Human Health: An Update for 2021. Nutrients.

[10] Moltu S.J., Nordvik T., Rossholt M.E., Wendel K., Chawla M., Server A., Gunnarsdottir G., Pripp A.H., Domellof M., Bratlie M. (2024). Arachidonic and docosahexaenoic acid supplementation and brain maturation in preterm infants; a double blind RCT. Clin. Nutr..

[11] Kelaiditis C.F., Gibson E.L., Dyall S.C. (2023). Effects of long-chain omega-3 polyunsaturated fatty acids on reducing anxiety and/or depression in adults; A systematic review and meta-analysis of randomised controlled trials. Prostaglandins Leukot. Essent. Fat. Acids.

[12] Shanab S.M.M., Hafez R.M., Fouad A.S. (2018). A review on algae and plants as potential source of arachidonic acid. J. Adv. Res..

[13] Jonasdottir S.H. (2019). Fatty Acid Profiles and Production in Marine Phytoplankton. Mar. Drugs.

[14] Guo F., Lee S.Y., Kainz M.J., Brett M.T. (2020). Fatty acids as dietary biomarkers in mangrove ecosystems: Current status and future perspective. Sci. Total Environ..

[15] Simopoulos A.P. (2016). An Increase in the Omega-6/Omega-3 Fatty Acid Ratio Increases the Risk for Obesity. Nutrients.

[16] Simopoulos A.P. (2000). Human Requirement for N-3 Polyunsaturated Fatty Acids. Poult. Sci..

[17] Gladyshev M.I., Sushchik N.N., Makhutova O.N. (2013). Production of EPA and DHA in aquatic ecosystems and their transfer to the land. Prostaglandins Other Lipid Mediat..

[18] Huang C.B., Ebersole J.L. (2010). A novel bioactivity of omega-3 polyunsaturated fatty acids and their ester derivatives. Mol. Oral Microbiol..

[19] Alsenani F., Tupally K.R., Chua E.T., Eltanahy E., Alsufyani H., Parekh H.S., Schenk P.M. (2020). Evaluation of microalgae and cyanobacteria as potential sources of antimicrobial compounds. Saudi Pharm. J..

[20] Peris-Martinez C., Pia-Ludena J.V., Rog-Revert M.J., Fernandez-Lopez E., Domingo J.C. (2023). Antioxidant and Anti-Inflammatory Effects of Oral Supplementation with a Highly-Concentrated Docosahexaenoic Acid (DHA) Triglyceride in Patients with Keratoconus: A Randomized Controlled Preliminary Study. Nutrients.

[21] Guo C.H., Hsia S.M., Chung C.H., Lin Y.C., Shih M.Y., Chen P.C., Peng C.L., Henning S.M., Hsu G.S.W., Li Z.P. (2021). Nutritional supplements in combination with chemotherapy or targeted therapy reduces tumor progression in mice bearing triple-negative breast cancer. J. Nutr. Biochem..

[22] Kumari P., Kumar M., Gupta V., Reddy C.R.K., Jha B. (2010). Tropical marine macroalgae as potential sources of nutritionally important PUFAs. Food Chem..

[23] Shoubaky G.A.E., Salem E.A.E.R. (2014). Active ingredients fatty acids as antibacterial agent from the brown algae Padina pavonica and Hormophysa triquetra. J. Coast. Life Med..

[24] Al-Saif S., Abdel-Raouf N., El-Wazanani H.A., Aref I.A. (2014). Antibacterial substances from marine algae isolated from Jeddah coast of Red sea, Saudi Arabia. Saudi J. Biol. Sci..

[25] Hixson S.M., Arts M.T. (2016). Climate warming is predicted to reduce omega-3, long-chain, polyunsaturated fatty acid production in phytoplankton. Glob. Chang. Biol..

[26] Holm Henry C., Fredricks Helen F., Bent Shavonna M., Lowenstein Daniel P., Ossolinski Justin E., Becker Kevin W., Johnson Winifred M., Schrage K., Van Mooy Benjamin A.S. (2022). Global ocean lipidomes show a universal relationship between temperature and lipid unsaturation. Science.

[27] Colombo S.M., Wacker A., Parrish C.C., Kainz M.J., Arts M.T. (2017). A fundamental dichotomy in long-chain polyunsaturated fatty acid abundance between and within marine and terrestrial ecosystems. Environ. Rev..

[28] Tittensor D.P., Mora C., Jetz W., Lotze H.K., Ricard D., Berghe E.V., Worm B. (2010). Global patterns and predictors of marine biodiversity across taxa. Nature.

[29] Kerswell A.P. (2006). Global biodiversity patterns of benthic marine algae. Ecology.

[30] Barton A.D., Dutkiewicz S., Flierl G., Bragg J., Follows M.J. (2010). Patterns of Diversity in Marine Phytoplankton. Science.

[31] Righetti D., Vogt M., Gruber N., Psomas A., Zimmermann N.E. (2019). Global pattern of phytoplankton diversity driven by temperature and environmental variability. Sci. Adv..

[32] Marzetz V., Koussoroplis A.M., Martin-Creuzburg D., Striebel M., Wacker A. (2017). Linking primary producer diversity and food quality effects on herbivores: A biochemical perspective. Sci. Rep..

[33] Young R.N. (1999). Importance of biodiversity to the modern pharmaceutical industry. Pure Appl. Chem..

[34] Chandini S., Ponesakki G., Suresh P.V., Bhaskar N. (2008). Seaweeds as a source of nutritionally beneficial compounds—A review. J. Food Sci. Technol. -Mysore-.

[35] Sonnewald M., El-Sherbiny M.M. (2017). Editorial: Red Sea biodiversity. Mar. Biodivers..

[36] Roberts C.M., McClean C.J., Veron J.E.N., Hawkins J.P., Allen G.R., McAllister D.E., Mittermeier C.G., Schueler F.W., Spalding M., Wells F. (2002). Marine Biodiversity Hotspots and Conservation Priorities for Tropical Reefs. Science.

[37] Berumen M.L., Voolstra C.R., Daffonchio D., Agusti S., Aranda M., Irigoien X., Jones B.H., Morán X.A.G., Duarte C.M., Voolstra C.R., Berumen M.L. (2019). The Red Sea: Environmental Gradients Shape a Natural Laboratory in a Nascent Ocean. Coral Reefs of the Red Sea.

[38] Manzelat S., Mufarrah A., Hasan A., Ali N. (2018). Macro algae of the Red Sea from Jizan, Saudi Arabia. Phykos.

[39] Galal El-Din Thabet Shams El-Din N., Rashedy S.H., Galal El-Din Thabet Shams El-Din N., Rashedy S.H. (2023). Biodiversity of Seaweeds in the Red Sea. Biodiversity of Seaweeds in the Egyptian Marine Waters: The Mediterranean Sea, Red Sea and Suez Canal.

[40] Prabowo D.A., Agusti S. (2019). Free-living dinoflagellates of the central Red Sea, Saudi Arabia: Variability, new records and potentially harmful species. Mar. Pollut. Bull..

[41] Nassar M.Z., Khairy H.M. (2014). Checklist of phytoplankton species in the Egyptian waters of the Red Sea and some surrounding habitats (1990–2010). Ann. Res. Rev. Biol..

[42] Angulo-Preckler C., Hempel C., Frappi S., Lim K.K., Terraneo T., Steinke D., Rabaoui L.J., Benzoni F., Duarte C.M. (2024). Unveiling biodiversity: The current status of marine species barcoding in Red Sea Metazoans. Glob. Ecol. Conserv..

[43] Chaidez V., Dreano D., Agusti S., Duarte C.M., Hoteit I. (2017). Decadal trends in Red Sea maximum surface temperature. Sci. Rep..

[44] Regaudie-de-Gioux A., Duarte C.M. (2012). Temperature dependence of planktonic metabolism in the ocean. Glob. Biogeochem. Cycles.

[45] Jin P., Gonzàlez G., Agustí S. (2020). Long-term exposure to increasing temperature can offset predicted losses in marine food quality (fatty acids) caused by ocean warming. Evol. Appl..

[46] Papina M., Meziane T., van Woesik R. (2003). Symbiotic zooxanthellae provide the host-coral Montipora digitata with polyunsaturated fatty acids. Comp. Biochem. Physiol. Part B Biochem. Mol. Biol..

[47] Allakhverdiev S.I., Nishiyama Y., Suzuki I., Tasaka Y., Murata N. (1999). Genetic engineering of the unsaturation of fatty acids in membrane lipids alters the tolerance of Synechocystis to salt stress. Proc. Natl. Acad. Sci. USA.

[48] Al-Adali K., Ahmed E., Kumar P., Ayaril N. (2012). Effect of Salinity, Temperature, Nutrients and CO_2_ on Growth of Two Species of Microalgae from Red Sea, Saudi Arabia. J. King Abdulaziz Univ.-Mar. Sci..

[49] Malibari R., Sayegh F., Elazzazy A.M., Baeshen M.N., Dourou M., Aggelis G. (2018). Reuse of shrimp farm wastewater as growth medium for marine microalgae isolated from Red Sea—Jeddah. J. Clean. Prod..

[50] Almutairi A.W. (2022). Evaluation of halophilic microalgae isolated from Rabigh Red Sea coastal area for biodiesel production: Screening and biochemical studies. Saudi J. Biol. Sci..

[51] Abomohra A.E., El-Naggar A.H., Alaswad S.O., Elsayed M., Li M., Li W. (2020). Enhancement of biodiesel yield from a halophilic green microalga isolated under extreme hypersaline conditions through stepwise salinity adaptation strategy. Bioresour. Technol..

[52] Pereira H., Barreira L., Custodio L., Alrokayan S., Mouffouk F., Varela J., Abu-Salah K.M., Ben-Hamadou R. (2013). Isolation and Fatty Acid Profile of Selected Microalgae Strains from the Red Sea for Biofuel Production. Energies.

[53] Hagar Kamal A., Samia H., Abdel-Hamied Mohammed R., Gihan Ahmed El S. (2023). Fatty acids composition and profiling of nine abundant marine Macroalgae, Egypt. GSC Biol. Pharm. Sci..

[54] EL-Shafay S.M. (2014). Biochemical Composition of Some Seaweed From Hurghada Coastal Along Red Sea Coastal, Egypt. Int. J. Basic Appl. Sci..

[55] Omar H.H., Abdullatif B.M., El-Kazan M.M., El-Gendy A.M. (2013). Red Sea Water and Biochemical Composition of Seaweeds at Southern Coast of Jeddah, Saudi Arabia. Life Sci. J..

[56] Kamal M., Abdel-Raouf N., Alwutayd K., AbdElgawad H., Abdelhameed M.S., Hammouda O., Elsayed K.N.M. (2023). Seasonal Changes in the Biochemical Composition of Dominant Macroalgal Species along the Egyptian Red Sea Shore. Biology.

[57] Al-Sofyani A.A., Niaz G.R. (2007). A comparative study of the components of the hard coral Seriatopora hystrix and the soft coral Xenia umbellata along the Jeddah coast, Saudi Arabia. Rev. De Biol. Mar. Y Oceanogr..

[58] Naggar M.E.E.E., El-Shora H.M., Shaaban-Dessouki S.A., Zaid A.M. (1995). Fatty acid composition of sargassum denticulatum and s. latifouum as in- fluenced by the time of collection and the plant organ. Qatar Univ. Sci. J..

[59] De Domenico S., De Rinaldis G., Mammone M., Bosch-Belmar M., Piraino S., Leone A. (2023). The Zooxanthellate Jellyfish Holobiont Cassiopea andromeda, a Source of Soluble Bioactive Compounds. Mar. Drugs.

[60] Imbs A.B., Yakovleva I.M., Pham L.Q. (2010). Distribution of lipids and fatty acids in the zooxanthellae and host of the soft coral *Sinularia* sp.. Fish. Sci..

[61] Mortillaro J.M., Pitt K.A., Lee S.Y., Meziane T. (2009). Light intensity influences the production and translocation of fatty acids by zooxanthellae in the jellyfish *Cassiopea* sp.. J. Exp. Mar. Biol. Ecol..

[62] Awai K., Matsuoka R., Shioi Y. Lipid and fatty acid compositions of Symbiodinium strains. Proceedings of the 12th International Coral Reef Symposium.

[63] Al-Mur B.A. (2021). Biological Activities of Avicennia marina Roots and Leaves Regarding Their Chemical Constituents. Arab. J. Sci. Eng..

[64] Sbrizzi S., Mitchell N., Campbell L.G., Arts M.T., Morris E., Borsato N., Colombo S.M. (2024). A phylogenetic approach for identifying new sources of economically important fatty acids in plants and algae. Plants People Planet.

[65] Bergé J.P., Barnathan G. (2005). Fatty acids from lipids of marine organisms: Molecular biodiversity, roles as biomarkers, biologically active compounds, and economical aspects. Adv. Biochem. Eng. Biotechnol..

[66] Montone C.M., Aita S.E., Catani M., Cavaliere C., Cerrato A., Piovesana S., Laganà A., Capriotti A.L. (2021). Profiling and quantitative analysis of underivatized fatty acids in Chlorella vulgaris microalgae by liquid chromatography-high resolution mass spectrometry. J. Sep. Sci..

[67] Hon G.M., Abel S., Smuts C.M., Jaarsveld P.v., Hassan M.S., Rensburg S.J.v., Erasmus R.T., Matsha T., Bekir S., Elikb A.K. (2012). Gas Chromatography Results Interpretation: Absolute Amounts Versus Relative Percentages. Gas Chromatography.

[68] Meziane T., Lee S.Y., Mfilinge P.L., Shin P.K.S., Lam M.H.W., Tsuchiya M. (2006). Inter-specific and geographical variations in the fatty acid composition of mangrove leaves: Implications for using fatty acids as a taxonomic tool and tracers of organic matter. Mar. Biol..

[69] Shilla D., Routh J. (2017). Using biochemical and isotopic tracers to characterise organic matter sources and their incorporation into estuarine food webs (Rufiji delta, Tanzania). Chem. Ecol..

[70] Alfaro A.C., Thomas F., Sergent L., Duxbury M. (2006). Identification of trophic interactions within an estuarine food web (northern New Zealand) using fatty acid biomarkers and stable isotopes. Estuar. Coast. Shelf Sci..

[71] Jiang Y., Fan K.-W., Tsz-Yeung Wong R., Chen F. (2004). Fatty Acid Composition and Squalene Content of the Marine Microalga Schizochytrium mangrovei. J. Agric. Food Chem..

[72] Fan K.W., Jiang Y., Faan Y.W., Chen F. (2007). Lipid characterization of mangrove thraustochytrid--Schizochytrium mangrovei. J. Agric. Food Chem..

[73] Beca-Carretero P., Guihéneuf F., Marín-Guirao L., Bernardeau-Esteller J., García-Muñoz R., Stengel D.B., Ruiz J.M. (2018). Effects of an experimental heat wave on fatty acid composition in two Mediterranean seagrass species. Mar. Pollut. Bull..

[74] Duarte B., Matos A.R., Pedro S., Marques J.C., Adão H., Caçador I. (2019). Dwarf eelgrass (Zostera noltii) leaf fatty acid profile during a natural restoration process: Physiological and ecological implications. Ecol. Indic..

[75] Abdel-Wahab M.A., El-Samawaty A.E.-R.M.A., Elgorban A.M., Bahkali A.H. (2022). Utilization of low-cost substrates for the production of high biomass, lipid and docosahexaenoic acid (DHA) using local native strain Aurantiochytrium sp. YB-05. J. King Saud Univ.—Sci..

[76] Waleed T.A., Abdel-Maksoud Y.K., Kanwar R.S., Sewilam H. (2024). Mangroves in Egypt and the Middle East: Current status, threats, and opportunities. Int. J. Environ. Sci. Technol..

[77] Bandaranayake W.M. (1998). Traditional and medicinal uses of mangroves. Mangroves Salt Marshes.

[78] Thatoi H., Samantaray D., Das S.K. (2016). The genus Avicennia, a pioneer group of dominant mangrove plant species with potential medicinal values: A review. Front. Life Sci..

[79] Ibrahim H.A.H., Abdel-Latif H.H., Zaghloul E.H. (2022). Phytochemical composition of Avicennia marina leaf extract, its antioxidant, antimicrobial potentials and inhibitory properties on Pseudomonas fluorescens biofilm. Egypt. J. Aquat. Res..

[80] Los D.A., Mironov K.S. (2015). Modes of Fatty Acid desaturation in cyanobacteria: An update. Life.

[81] Post A.F., Dedej Z., Gottlieb R., Li H., Thomas D.N. (2002). Spatial and temporal distribution of Trichodesmium spp. in the stratified Gulf of Aqaba, Red Sea. Mar. Ecol. Prog. Ser..

[82] Tocher D., Leaver M., Hodgson P. (1998). Recent advances in the biochemistry and molecular biology of fatty acyl desaturases. Prog. Lipid Res..

[83] Santos-Merino M., Gutiérrez-Lanza R., Nogales J., García J.L., de la Cruz F. (2022). Synechococcus elongatus PCC 7942 as a Platform for Bioproduction of Omega-3 Fatty Acids. Life.

[84] Patil V., Källqvist T., Olsen E., Vogt G., Gislerød H.R. (2007). Fatty acid composition of 12 microalgae for possible use in aquaculture feed. Aquac. Int..

[85] Holton R.W., Blecker H.H., Stevens T.S. (1968). Fatty Acids in Blue-Green Algae: Possible Relation to Phylogenetic Position. Science.

[86] Carpenter E.J., Harvey H.R., Fry B., Capone D.G. (1997). Biogeochemical tracers of the marine cyanobacterium Trichodesmium. Deep Sea Res. Part I Oceanogr. Res. Pap..

[87] Parker P.L., Van Baalen C., Maurer L. (1967). Fatty Acids in Eleven Species of Blue-Green Algae: Geochemical Significance. Science.

[88] Leu E., Wängberg S.-Å., Wulff A., Falk-Petersen S., Børre Ørbæk J., Hessen D.O. (2006). Effects of changes in ambient PAR and UV radiation on the nutritional quality of an Arctic diatom (Thalassiosira antarctica var. borealis). J. Exp. Mar. Biol. Ecol..

[89] Shishlyannikov S.M., Klimenkov I.V., Bedoshvili Y.D., Mikhailov I.S., Gorshkov A.G. (2014). Effect of mixotrophic growth on the ultrastructure and fatty acid composition of the diatom Synedra acus from Lake Baikal. J. Biol. Res..

[90] Almeyda M.D., Scodelaro Bilbao P.G., Popovich C.A., Constenla D., Leonardi P.I. (2020). Enhancement of polyunsaturated fatty acid production under low-temperature stress in Cylindrotheca closterium. J. Appl. Phycol..

[91] Salvador López J.M., Vandeputte M., Van Bogaert I.N.A. (2022). Oleaginous yeasts: Time to rethink the definition?. Yeast.

[92] De Rinaldis G., Leone A., De Domenico S., Bosch-Belmar M., Slizyte R., Milisenda G., Santucci A., Albano C., Piraino S. (2021). Biochemical Characterization of Cassiopea andromeda (Forsskål, 1775), Another Red Sea Jellyfish in the Western Mediterranean Sea. Mar. Drugs.

[93] Jamarun N., Pazla R., Jayanegara A., Yanti G. (2020). Chemical composition and rumen fermentation profile of mangrove leaves (Avicennia marina) from West Sumatra, Indonesia. Biodiversitas J. Biol. Divers..

[94] Guschina I.A., Harwood J.L. (2009). Algal lipids and effect of the environment on their biochemistry. Lipids in Aquatic Ecosystems.

[95] Galloway A.W., Winder M. (2015). Partitioning the Relative Importance of Phylogeny and Environmental Conditions on Phytoplankton Fatty Acids. PLoS ONE.

[96] Lowndes A.G. (1942). Percentage of Water in Jelly-Fish. Nature.

[97] Oku H., Baba S., Koga H., Takara K., Iwasaki H. (2003). Lipid composition of mangrove and its relevance to salt tolerance. J. Plant Res..

[98] Brown M.R., Dunstan G.A., Norwood S.J., Miller K.A. (1996). Effects of harvest stage and light on the biochemical composition of the diatom Thalassiosira pseudonana 1. J. Phycol..

[99] Taipale S., Peltomaa E., Salmi P. (2020). Variation in omega-3 and omega-6 Polyunsaturated Fatty Acids Produced by Different Phytoplankton Taxa at Early and Late Growth Phase. Biomolecules.

[100] Tsai C.H., Zienkiewicz K., Amstutz C.L., Brink B.G., Warakanont J., Roston R., Benning C. (2015). Dynamics of protein and polar lipid recruitment during lipid droplet assembly in *Chlamydomonas reinhardtii*. Plant J..

[101] Weng L.-C., Pasaribu B., Ping Lin I., Tsai C.-H., Chen C.-S., Jiang P.-L. (2014). Nitrogen Deprivation Induces Lipid Droplet Accumulation and Alters Fatty Acid Metabolism in Symbiotic Dinoflagellates Isolated from *Aiptasia pulchella*. Sci. Rep..

[102] Hazel J.R. (1995). Thermal Adaptation in Biological Membranes: Is Homeoviscous Adaptation the Explanation?. Annu. Rev. Physiol..

[103] Sinensky M. (1974). Homeoviscous adaptation—A homeostatic process that regulates the viscosity of membrane lipids in *Escherichia coli*. Proc. Natl. Acad. Sci. USA.

[104] Crossland C.J., Barnes D.J., Borowitzka M.A. (1980). Diurnal lipid and mucus production in the staghorn coral *Acropora acuminata*. Mar. Biol..

[105] Liu C., Zhang Y., Huang L., Yu X., Luo Y., Jiang L., Sun Y., Liu S., Huang H. (2022). Differences in Fatty Acids and Lipids of Massive and Branching Reef-Building Corals and Response to Environmental Changes. Front. Mar. Sci..

[106] Imbs A.B., Dembitsky V.M. (2023). Coral Lipids. Mar. Drugs.

[107] Ward S. (1995). Two patterns of energy allocation for growth, reproduction and lipid storage in the scleractinian coral *Pocillopora damicornis*. Coral Reefs.

[108] Osman E.O., Suggett D.J., Attalla T.M., Casartelli M., Cook N., El-Sadek I., Gallab A., Goergen E.A., Garcias-Bonet N., Glanz J.S. (2024). Spatial variation in spawning timing for multi-species *Acropora* assemblages in the Red Sea. Front. Mar. Sci..

[109] Parkes R., Archer L., Gee D.M., Smyth T.J., Gillespie E., Touzet N. (2021). Differential responses in EPA and fucoxanthin production by the marine diatom Stauroneis sp. under varying cultivation conditions. Biotechnol. Prog..

[110] Thompson P.A., Guo M.-x., Harrison P.J., Whyte J.N.C. (1992). Effects of variation in temperature. II. On the fatty acid composition of eight species of marine phytoplankton1. J. Phycol..

[111] Peltomaa E., Hällfors H., Taipale S.J. (2019). Comparison of Diatoms and Dinoflagellates from Different Habitats as Sources of PUFAs. Mar Drugs.

[112] Remize M., Planchon F., Loh A.N., Le Grand F., Bideau A., Goïc N., Fleury E., Miner P., Corvaisier R., Volety A. (2020). Study of Synthesis Pathways of the Essential Polyunsaturated Fatty Acid 20:5n-3 in the Diatom *Chaetoceros Muelleri* Using 13C-Isotope Labeling. Biomolecules.

[113] Kelly J.R., Scheibling R.E. (2012). Fatty acids as dietary tracers in benthic food webs. Mar. Ecol. Prog. Ser..

[114] Budge S.M., Devred E., Forget M.-H., Stuart V., Trzcinski M.K., Sathyendranath S., Platt T. (2014). Estimating concentrations of essential omega-3 fatty acids in the ocean: Supply and demand. ICES J. Mar. Sci..

[115] Mai T.D., Lee-Chang K.J., Jameson I.D., Hoang T., Cai N.B.A., Pham H.Q. (2021). Fatty Acid Profiles of Selected Microalgae Used as Live Feeds for Shrimp Postlarvae in Vietnam. Aquac. J..

[116] Liu F., Wang P., Xiong X., Zeng X., Zhang X., Wu G. (2021). A Review of Nervonic Acid Production in Plants: Prospects for the Genetic Engineering of High Nervonic Acid Cultivars Plants. Front. Plant Sci..

[117] Fan Y., Meng H.-M., Hu G.-R., Li F.-L. (2018). Biosynthesis of nervonic acid and perspectives for its production by microalgae and other microorganisms. Appl. Microbiol. Biotechnol..

[118] Al-Hammady M.A.M. (2013). The effect of zooxanthellae availability on the rates of skeletal growth in the Red Sea coral *Acropora hemprichii*. Egypt. J. Aquat. Res..

[119] Kabeya N., Fonseca Miguel M., Ferrier David E.K., Navarro Juan C., Bay Line K., Francis David S., Tocher Douglas R., Castro L.F.C., Monroig Ó. (2018). Genes for de novo biosynthesis of omega-3 polyunsaturated fatty acids are widespread in animals. Sci. Adv..

[120] Monroig Ó., Kabeya N. (2018). Desaturases and elongases involved in polyunsaturated fatty acid biosynthesis in aquatic invertebrates: A comprehensive review. Fish. Sci..

[121] Steinberg C.E.W., Steinberg C.E.W. (2022). Biosynthesis of Polyunsaturated Fatty Acids—‘Many Can, Some Can’t’. Aquatic Animal Nutrition: Organic Macro- and Micro-Nutrients.

[122] Garrido D., Kabeya N., Hontoria F., Navarro J.C., Reis D.B., Martín M.V., Rodríguez C., Almansa E., Monroig Ó. (2019). Methyl-end desaturases with ∆12 and ω3 regioselectivities enable the de novo PUFA biosynthesis in the cephalopod Octopus vulgaris. Biochim. Et Biophys. Acta (BBA)—Mol. Cell Biol. Lipids.

[123] Safuan C.D.M., Tan H.S., Samshuri M.A., Afiq-Firdaus A.M., Bachok Z. (2023). Chemotaxonomy of reef building corals (family: Acroporidae) via fatty acid biomarkers. Biochem. Syst. Ecol..

[124] Al-Adilah H., Al-Sharrah T.K., Al-Bader D., Ebel R., Küpper F.C., Kumari P. (2021). Assessment of Arabian Gulf Seaweeds from Kuwait as Sources of Nutritionally Important Polyunsaturated Fatty Acids (PUFAs). Foods.

[125] Silva G., Pereira R.B., Valentão P., Andrade P.B., Sousa C. (2013). Distinct fatty acid profile of ten brown macroalgae. Rev. Bras. De Farmacogn..

[126] Van Ginneken V.J., Helsper J.P., de Visser W., van Keulen H., Brandenburg W.A. (2011). Polyunsaturated fatty acids in various macroalgal species from north Atlantic and tropical seas. Lipids Health Dis..

[127] Susanto E., Fahmi A.S., Abe M., Hosokawa M., Miyashita K. (2016). Lipids, Fatty Acids, and Fucoxanthin Content from Temperate and Tropical Brown Seaweeds. Aquat. Procedia.

[128] Matanjun P., Mohamed S., Mustapha N.M., Muhammad K. (2009). Nutrient content of tropical edible seaweeds, *Eucheuma cottonii*, *Caulerpa lentillifera* and, *Sargassum polycystum*. J. Appl. Phycol..

[129] Kumari P., Bijo A.J., Mantri V.A., Reddy C.R.K., Jha B. (2013). Fatty acid profiling of tropical marine macroalgae: An analysis from chemotaxonomic and nutritional perspectives. Phytochemistry.

[130] Feijão E., Franzitta M., Cabrita M.T., Caçador I., Duarte B., Gameiro C., Matos A.R. (2020). Marine heat waves alter gene expression of key enzymes of membrane and storage lipids metabolism in *Phaeodactylum tricornutum*. Plant Physiol. Biochem..

[131] Hu X., Tang X., Bi Z., Zhao Q., Ren L. (2021). Adaptive evolution of microalgae Schizochytrium sp. under high temperature for efficient production of docosahexaeonic acid. Algal Res..

[132] Burdock G.A., Carabin I.G. (2004). Generally recognized as safe (GRAS): History and description. Toxicol. Lett..

[133] Cole J.J. (1982). Interactions Between Bacteria and Algae in Aquatic Ecosystems. Annu. Rev. Ecol. Evol. Syst..

[134] Aljbour S.M., Agustí S. (2024). Illuminating Cassiopea jellyfish: Biochemical revelations from metabolism to coloration under ultraviolet A and photosynthetically active radiation. Front. Mar. Sci..

[135] Tomas C.E. (1997). Identifying Marine Phytoplankton.

[136] Sonnenberg R., Nolte A.W., Tautz D. (2007). An evaluation of LSU rDNA D1-D2 sequences for their use in species identification. Front. Zool..

[137] Guillard R.R.L., Ryther J.H. (1962). Studies of marine planktonic diatoms: I. Cyclotella nana hustedt, and detonula confervacea (cleve) gran. Can. J. Microbiol..

[138] Lewis T., Nichols P.D., McMeekin T.A. (2000). Evaluation of extraction methods for recovery of fatty acids from lipid-producing microheterotrophs. J. Microbiol. Methods.

[139] Rey F., Melo T., Lopes D., Couto D., Marques F., Domingues M.R. (2022). Applications of lipidomics in marine organisms: Progress, challenges and future perspectives. Mol. Omics.

[140] Couturier L.I.E., Michel L.N., Amaro T., Budge S.M., da Costa E., De Troch M., Di Dato V., Fink P., Giraldo C., Le Grand F. (2020). State of art and best practices for fatty acid analysis in aquatic sciences. ICES J. Mar. Sci..

[141] Simopoulos A.P. (2002). The importance of the ratio of omega-6/omega-3 essential fatty acids. Biomed. Pharmacother..

[142] Kainz M., Arts M.T., Mazumder A. (2004). Essential fatty acids in the planktonic food web and their ecological role for higher trophic levels. Limnol. Oceanogr..

