# Supplementary material for: Photobiota of the Tropical Red Sea: Fatty Acid Profile Analysis and Nutritional Quality Assessments
Source: Molecules. 2025 Jan 31;30(3):621. doi: 10.3390/molecules30030621 (PMC11820627; doi:10.3390/molecules30030621)
Supplement: Supplementary file 1 [file molecules-30-00621-s001.zip › molecules-3369667-supplementary.pdf]

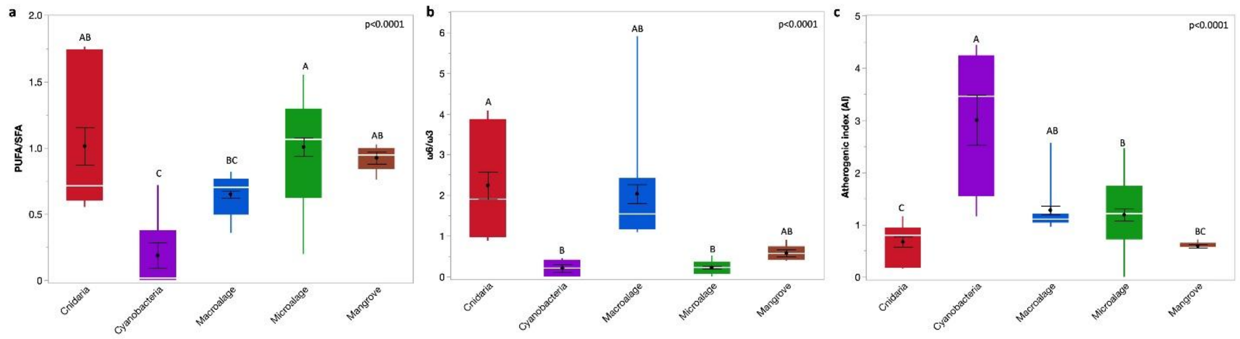

Figure S1. Comparative boxplot of FA quality indexes illustrating Red Sea photobiota group variations, in (a) Polyunsaturated FA/saturated FA (PUFA/SFA) ratio, (b) ω6/ω3 ratio, and (c) atherogenic index (AI). Box edges represent the first and third quartiles (Q1 and Q3), whiskers extend to the minimum and maximum values. Horizontal lines indicate medians. Black dot and vertical line indicate mean and standard error. Statistical analyses: Kruskal-Wallis test followed by Dunn test (groups that do not share a letter are significantly different).

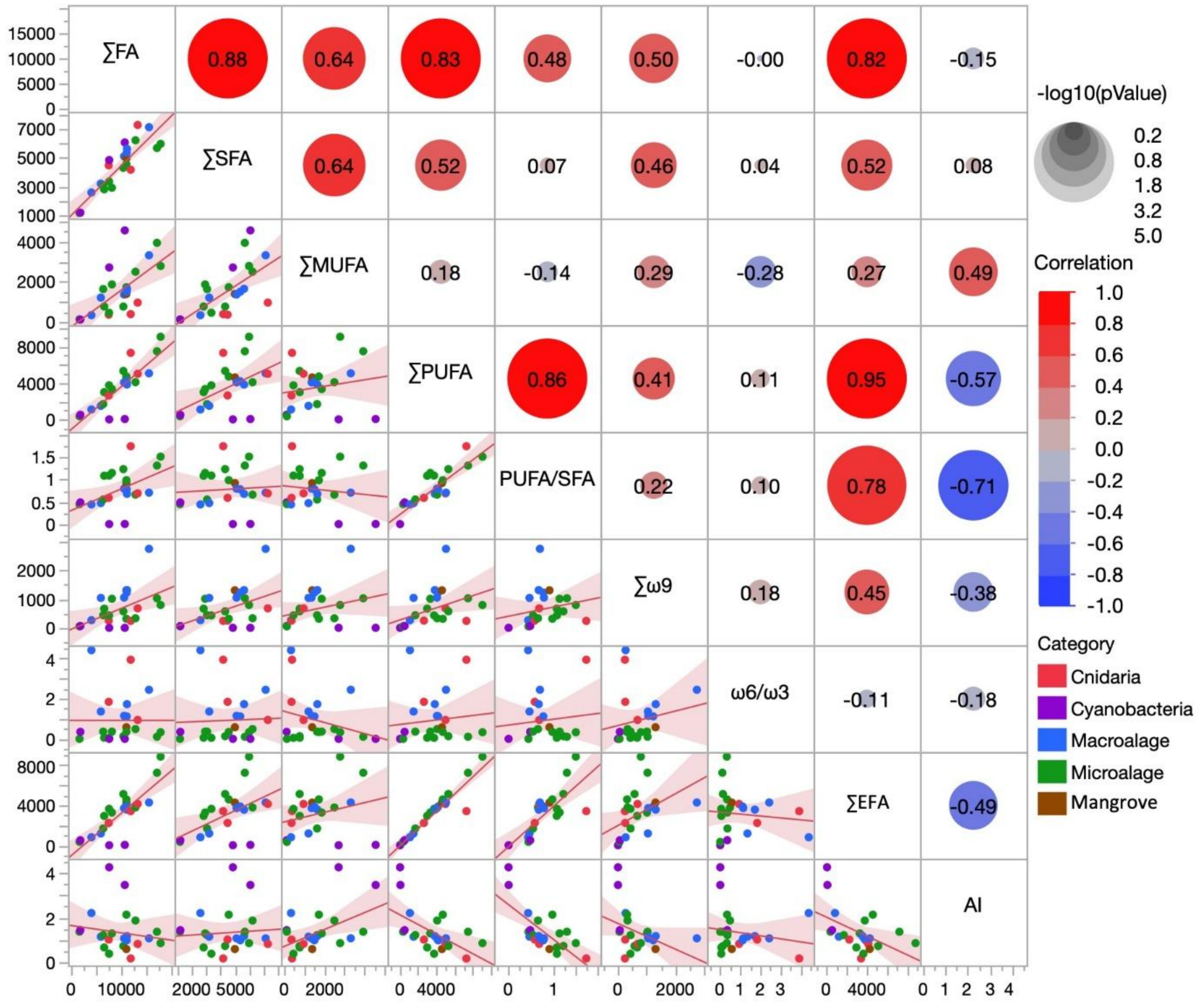

Figure S2. Correlation matrix of FA quality indexes across Red Sea photobiota groups. Points in scatter plots are means of replicates. FA, Fatty acid; PUFA, polyunsaturated FA; MUFA, monounsaturated FA; SFA, saturated FA; EFAs, essential FAs; AI, atherogenic index; PUFA/SFA, polyunsaturated FA/saturated FA ratio.

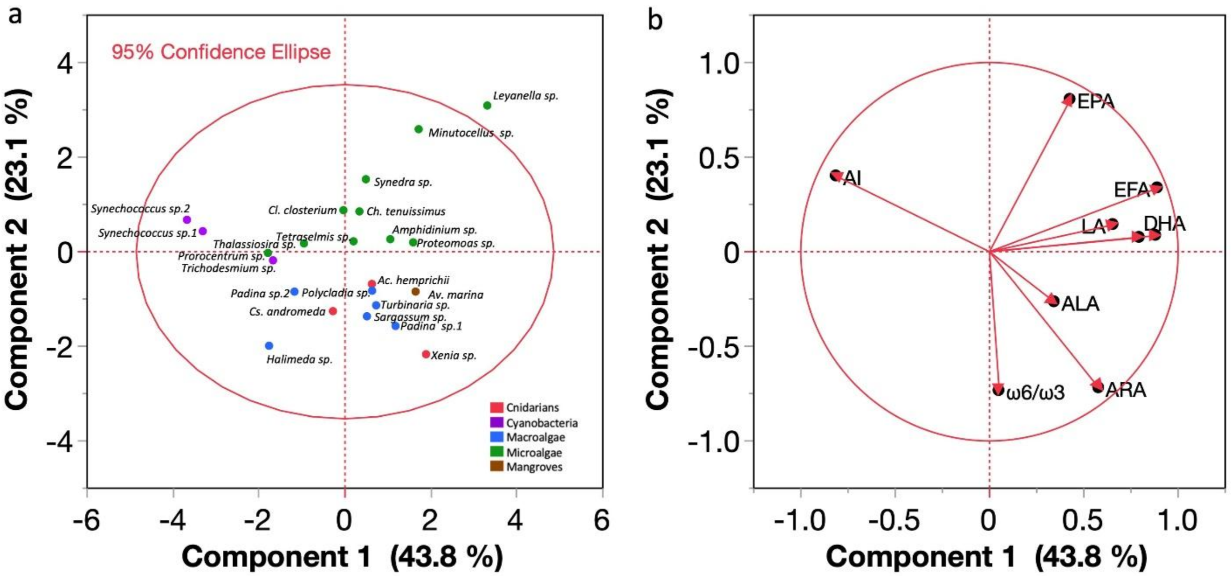

Figure S3. Principal component analysis of Red Sea photobiota based on their FA nutritional quality. (a) score plot illustrating FA nutritional quality across the 23 photosynthetic organisms. (b) loading plot displays eicosapentaenoic acid (EPA), docosahexaenoic acid (DHA), alpha-linolenic acid (ALA), linolenic acid (LA), and arachidonic acid (ARA) and indexes ω6/ω3 ratio, polyunsaturated FA/saturated ratio (PUFA/SFA), atherogenic index (AI), and sum (EFAs).

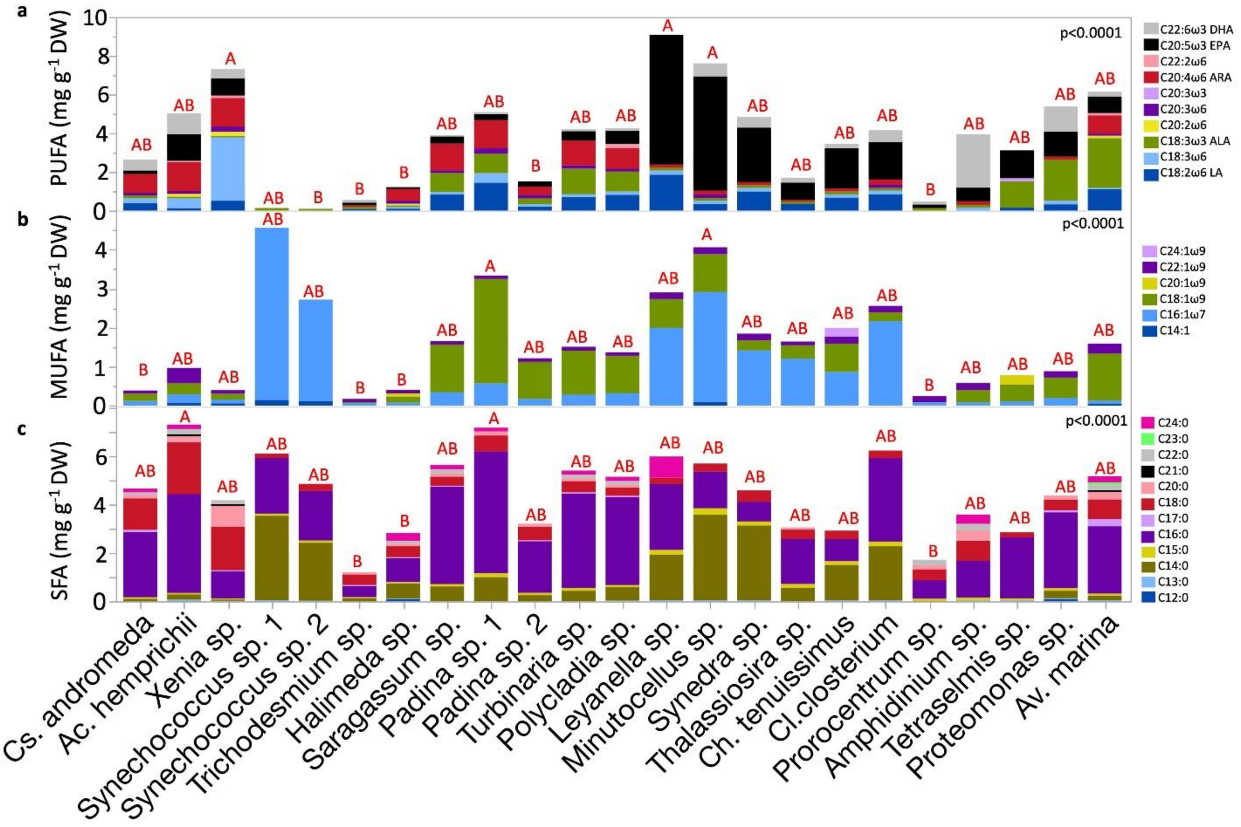

Figure S4. Distribution of FA content according to saturation across species (**a**) polyunsaturated FA (PUFA), **(b)** monounsaturated FA (MUFA), and (c) saturated FA (SFA) contents. Bars represent means, mg g^{-1} DW. Statistical analyses: Kruskal-Wallis test followed by Dunn test, where means that do not share a letter are significantly different. FA, Fatty acid; DW, dry weight.

Table S1. FA quality indexes of Red Sea organisms. Mean ± Standard error (mg g^{-1} DW); NA denotes "not available". SFA, saturated FA; MUFA, monounsaturated FA; PUFA, polyunsaturated FA; PUFA/SFA, polyunsaturated FA/saturated FA ratio; EFAs, essential FAs; AI, atherogenic index.

	∑SFA	∑MUFA	∑PUFA	PUFA/SFA	∑ω3	∑ω6	∑ω9	ω6/ω3	∑ EFA	AI
Xenia sp.	4.179 ± 0.05	0.395 ± 0.00	7.312 ± 0.09	0.002 ± 0.00	1.429 ± 0.03	5.622 ± 0.08	0.242 ± 0.00	0.004 ± 0.00	3.424 ± 0.05	0.000 ± 0.00
Ac. hemprichii	7.298 ± 0.73	0.966 ± 0.09	5.017 ± 0.45	0.001 ± 0.00	2.522 ± 0.25	2.359 ± 0.21	0.680 ± 0.07	0.001 ± 0.00	4.142 ± 0.38	0.001 ± 0.00
Cs. andromeda	4.487 ± 0.39	0.369 ± 0.04	2.645 ± 0.18	0.001 ± 0.00	0.894 ± 0.09	1.624 ± 0.10	0.252 ± 0.03	0.002 ± 0.00	2.254 ± 0.15	0.001 ± 0.00
Synechococcus sp. 1	6.080 ± 2.07	4.564 ± 1.34	0.066 ± 0.07	0.000 ± 0.00	0.066 ± 0.07	0.000 ± 0.00	0.000 ± 0.00	0.000 ± 0.00	0.066 ± 0.07	0.003 ± 0.00
Synechococcus sp. 2	4.856 ± 0.46	2.718 ± 0.35	0.030 ± 0.03	0.000 ± 0.00	0.030 ± 0.03	0.000 ± 0.00	0.000 ± 0.00	0.000 ± 0.00	0.030 ± 0.03	0.004 ± 0.00
Trichodesmium sp.	1.186 ± 0.31	0.124 ± 0.02	0.528 ± 0.08	0.000 ± 0.00	0.390 ± 0.05	0.139 ± 0.03	0.064 ± 0.02	0.000 ± 0.00	0.528 ± 0.08	0.001 ± 0.00
Halimeda sp.	2.590 ± 0.41	0.343 ± 0.03	1.114 ± 0.10	0.000 ± 0.00	0.179 ± 0.00	0.787 ± 0.09	0.266 ± 0.03	0.004 ± 0.00	0.825 ± 0.06	0.002 ± 0.00
Saragassum sp.	5.638 ± 0.09	1.656 ± 0.04	3.847 ± 0.06	0.001 ± 0.00	1.360 ± 0.02	2.348 ± 0.04	1.315 ± 0.03	0.002 ± 0.00	3.576 ± 0.06	0.001 ± 0.00
Padina sp.1	7.145 ± 0.11	3.322 ± 0.08	5.071 ± 0.09	0.001 ± 0.00	1.395 ± 0.05	3.396 ± 0.05	2.753 ± 0.08	0.002 ± 0.00	4.290 ± 0.07	0.001 ± 0.00
Padina sp. 2	3.206 ± 0.25	1.214 ± 0.08	1.511 ± 0.08	0.000 ± 0.00	0.568 ± 0.03	0.771 ± 0.04	1.043 ± 0.07	0.001 ± 0.00	1.207 ± 0.07	0.001 ± 0.00
Polycladia sp.	5.114 ± 0.10	1.369 ± 0.01	4.084 ± 0.10	0.001 ± 0.00	1.839 ± 0.04	2.108 ± 0.06	1.055 ± 0.01	0.001 ± 0.00	3.730 ± 0.06	0.001 ± 0.00
Turbinaria sp.	5.370 ± 0.16	1.509 ± 0.02	4.207 ± 0.05	0.001 ± 0.00	1.906 ± 0.02	2.132 ± 0.03	1.229 ± 0.02	0.001 ± 0.00	3.884 ± 0.04	0.001 ± 0.00
Prorocentrum sp.	1.164 ± 0.34	0.136 ± 0.05	0.368 ± 0.05	0.000 ± 0.00	0.368 ± 0.05	0.000 ± 0.00	0.054 ± 0.05	0.000 ± 0.00	0.368 ± 0.05	0.001 ± 0.00
Leyanella sp.	5.985 ± 0.70	2.791 ± 0.25	9.074 ± 1.08	0.002 ± 0.00	6.838 ± 0.85	2.236 ± 0.23	0.796 ± 0.02	0.000 ± 0.00	8.854 ± 1.07	0.001 ± 0.00
Minutocellus sp.	5.693 ± 0.05	3.943 ± 0.07	7.488 ± 0.15	0.001 ± 0.00	6.714 ± 0.09	0.713 ± 0.01	1.030 ± 0.08	0.000 ± 0.00	7.249 ± 0.09	0.001 ± 0.00
Synedra sp.	4.569 ± 1.06	1.731 ± 0.51	4.721 ± 1.73	0.001 ± 0.00	3.500 ± 1.29	1.221 ± 0.44	0.317 ± 0.07	0.000 ± 0.00	4.599 ± 1.67	0.002 ± 0.00
Thalassiosira sp.	3.054 ± 0.35	1.643 ± 0.16	1.703 ± 0.23	0.001 ± 0.00	1.251 ± 0.16	0.452 ± 0.06	0.440 ± 0.05	0.000 ± 0.00	1.703 ± 0.23	0.001 ± 0.00
Tetraselmis sp.	2.813 ± 0.29	0.777 ± 0.02	3.006 ± 0.08	0.001 ± 0.00	2.849 ± 0.08	0.158 ± 0.00	0.669 ± 0.02	0.000 ± 0.00	2.952 ± 0.13	0.001 ± 0.00
Amphidinium sp.	3.354 ± 0.28	0.461 ± 0.10	3.760 ± 1.02	0.001 ± 0.00	3.591 ± 0.96	0.169 ± 0.10	0.434 ± 0.07	0.000 ± 0.00	3.652 ± 1.02	0.000 ± 0.00
Ch. tenuissimus	2.920 ± 0.43	1.872 ± 0.22	3.346 ± 0.47	0.001 ± 0.00	2.460 ± 0.36	0.885 ± 0.12	1.012 ± 0.12	0.000 ± 0.00	3.292 ± 0.46	0.001 ± 0.00
Cl.closterium	6.231 ± 0.41	2.503 ± 0.12	4.102 ± 0.15	0.001 ± 0.00	2.682 ± 0.09	1.311 ± 0.01	0.334 ± 0.06	0.000 ± 0.00	3.797 ± 0.10	0.002 ± 0.00
Proteomonas sp.	4.313 ± 0.46	0.772 ± 0.11	5.332 ± 0.60	0.001 ± 0.00	4.707 ± 0.52	0.625 ± 0.09	0.576 ± 0.10	0.000 ± 0.00	5.135 ± 0.59	0.001 ± 0.00
Av. marina	4.995 ± 0.17	1.398 ± 0.20	4.624 ± 0.30	0.001 ± 0.00	2.992 ± 0.33	1.611 ± 0.07	1.308 ± 0.23	0.001 ± 0.00	4.279 ± 0.38	0.001 ± 0.00

Table S2. List of studied organisms, sampling location, type of the sample, number of replicates, amount analyzed, and isolation and analysis dates.

Phylum	Species/Strain	sampling location	Type	Isolation date[#]	Analysis date	Weight analyzed (mean, mg DW)
Bacillariophyta	*Cylindrotheca closterium*	22.4331 N, 38.9974 E	Mono-specific culture*	March 2015	September 2023	25.00 (n=3)
Bacillariophyta	*Thalassiosira* sp.	22.2528°N, 38.9612°E	Mono-specific culture*	August 2015	August 2023	47.40 (n=3)
Bacillariophyta	*Chaetoceros tenuissimus*	22.2528°N, 38.9612°E	Mono-specific culture*	August 2015	September 2023	25.00 (n=3)
Bacillariophyta	*Leyanella* sp.	22.4331 N, 38.9974 E	Mono-specific culture*	March 2015	September 2023	25.00 (n=3)
Bacillariophyta	*Synedra* sp.	22.2528°N, 38.9612°E	Mono-specific culture*	March 2015	September 2023	25.00 (n=3)
Bacillariophyta	*Minutocellus* sp.	22.4331 N, 38.9974 E	Mono-specific culture*	March 2015	September 2023	25.00 (n=3)
Dinophyta	*Prorocentrum* sp.	22.4331 N, 38.9974 E	Mono-specific culture*	July 2016	September 2023	28.05 (n=3)
Dinophyta	*Amphidinium* sp.	22.4331 N, 38.9974 E	Mono-specific culture*	July 2016	September 2023	25.00 (n=3)
Cryptophyta	*Proteomonas* sp.	22.4331 N, 38.9974 E	Mono-specific culture*	March 2015	September 2023	25.00 (n=3)
Chlorophyta	*Tetraselmis* sp.	22.4331 N, 38.9974 E	Mono-specific culture*	March 2015	September 2023	25.00 (n=3)
Cyanophyta	*Synechococcus* sp. strain 1	22.3093 N, 38.9974 E	Mono-specific culture*	July 2017	September 2023	42.10 (n=2)
Cyanophyta	*Synechococcus* sp. strain 2	22.3093 N, 38.9974 E	Mono-specific culture*	July 2017	September 2023	41.94 (n=3)
Cyanophyta	*Trichodesmium* sp. bloom	22.2030 N, 39.0513 E	Natural	late May 2022	August 2023	51.32 (n=3)
Cnidaria	*Acropora hemprichii*	22.2528°N, 38.9612°E	Aquaculture	2021	September 2023	50.10 (n=5)
Cnidaria	*Xenia* sp.	22.2528°N, 38.9612°E	Aquaculture	2021	September 2023	50.04 (n=5)
Cnidaria	*Cassiopea andromeda*	22.2030 N, 39.0513 E	Natural	June 2023	August 2023	50.00 (n=5)
Rhizophora	*Avicennia marina*	22.2030 N, 39.0513 E	Natural	July 2023	August 2023	50.00 (n=5)
Phaeophyta	*Padina* sp. strain 2	22.2724 N, 39.0456 E	Natural	April 2022	August 2023	50.16 (n=5)
Chlorophyta	*Halimeda* sp.	19.3150 N, 40.0151E	Natural	April 2022	September 2023	50.34 (n=5)
Phaeophyta	*Sargassum* sp.	22.2030 N, 39.0513 E	Natural	December 2022	August 2023	50.00 (n=5)
Phaeophyta	*Turbinaria* sp.	22.2030 N, 39.0513 E	Natural	December 2022	August 2023	50.00 (n=5)
Phaeophyta	*Polycladia* sp.	22.2030 N, 39.0513 E	Natural	December 2022	September 2023	50.06 (n=5)
Phaeophyta	*Padina* sp. strain 1	22.2030 N, 39.0513 E	Natural	June 2023	August 2023	50.08 (n=5)

#Isolation date, which is not nesessrily the same date at which the sample was isolated for FA analysis, indicate the initial isolation of the organism from the Red Sea water. *Cultures isolated from the original stock cultures and grown in triplicates at August-September 2023. * Cultures harvested at early exponential phase.
